# Patient overlap, cohort-relative scaling, and biological context in cross-cohort evaluation of a mitochondrial proteostasis-associated score in triple-negative breast cancer

**DOI:** 10.1371/journal.pone.0355652

**Published:** 2026-10-09

**Authors:** Xiaoyuan Weng, Yanqi Tang, Caiji Wang, Zhaofeng Huang, Xiyin Huang, Jinsheng Wang

**Affiliations:** 1 Department of Clinical Medicine, Quanzhou Medical College, Quanzhou, Fujian, China; 2 Department of Breast Surgery, Second Affiliated Hospital of Fujian Medical University, Quanzhou, Fujian, China; 3 Medical Research Center of Quanzhou Medical College, Quanzhou Medical College, Quanzhou, Fujian, China; 4 Thoracic Surgery Department, People’s Hospital Affiliated to Quanzhou Medical College, Quanzhou, Fujian, China; Michigan Medicine, UNITED STATES OF AMERICA

## Abstract

**Background:**

Public transcriptomic cohort evaluation can be distorted by patient reuse, dependence among study sources, and cohort-relative normalization. Objective: To evaluate the association between a fixed 15-gene cohort-relative mitochondrial proteostasis score and source-study-defined pathological complete response (pCR) across four accession-defined cohorts from three principal study sources.

**Methods:**

The fixed score comprised 15 genes with equal positive weights, a specified expected direction, and a platform-coverage rule. After patient-level deduplication and duplicate-aliquot collapse, 285 patients with strict triple-negative breast cancer, including 104 with pCR, were analyzed. Cohort-specific odds ratios (ORs) per one-standard-deviation score increase were pooled by restricted maximum-likelihood random-effects meta-analysis with modified Hartung-Knapp inference. Sensitivity analyses summarized three study sources, used Paule-Mandel heterogeneity estimation, and applied one fixed single-sample RankScore implementation.

**Results:**

The primary four-cohort estimate was OR 0.958 (95% CI, 0.639–1.437; P = 0.760; tau^2 = 0). The three-source REML estimate was OR 0.971 (95% CI, 0.492–1.914; P = 0.868; tau^2 = 0.0201). The RankScore sensitivity was imprecise (OR 0.613 per 0.10 RankScore; 95% CI, 0.148–2.542; P = 0.354). The overlap assessment identified 57 strict-TNBC patients duplicated between GSE20194 and GSE25055; treating them as an additional accession effect increased nominal n from 285 to 342 without adding independent participants. Contextual NeoTRIP, single-cell, and I-SPY2 analyses were included to characterize biological and treatment context.

**Conclusions:**

Across four accession-defined cohorts from three principal study sources, the evaluated score implementations did not show a reproducible association with pCR. Source-level estimates remained imprecise. Patient overlap, reference dependence, and limited independent study sources are key considerations in cross-cohort biomarker evaluation.

## Introduction

Pathological complete response (pCR) after neoadjuvant systemic therapy is associated with improved long-term outcomes in triple-negative breast cancer (TNBC), but a molecular correlate observed in one dataset is not automatically a clinically useful or broadly generalizable predictor [1,2]. A candidate biomarker should be evaluated across independent patients and study sources, and its interpretation should account for platform, treatment, specimen-processing, and broad biological programs such as proliferation and tissue composition.

Public expression repositories make multi-cohort evaluation possible, but accession numbers are administrative identifiers rather than evidence of participant independence. Reuse of the same patients across accessions can double-count observations, place the same individuals in nominally separate cohorts, and narrow uncertainty without adding biological information. A second challenge is that cohort-specific standardization can make a transcriptomic score reproducibly calculable while leaving it dependent on the reference distribution used for scoring.

MitoCarta3.0 provides manually curated mitochondrial pathway annotations, including chaperone and protein-quality-control functions [3]. Chemotherapy-surviving TNBC models have shown adaptive increases in mitochondrial oxidative phosphorylation and mitochondrial fusion [4]; chaperones and protein-quality-control systems maintain mitochondrial proteome function under stress, providing a biological rationale to examine mitochondrial proteostasis in relation to treatment response. We used the MitoCarta3.0 Chaperones pathway to define an equally weighted 15-gene candidate score.

We evaluated whether the fixed 15-gene cohort-relative score was associated with pCR across non-overlapping TNBC cohorts and examined the consistency and interpretation of that association across study sources. The analysis included patient-level overlap assessment, cross-cohort meta-analysis, a single-sample scoring sensitivity, and longitudinal and single-cell analyses of biological context.

## Materials and methods

### Study design, estimand, and reporting

This retrospective, multi-cohort study used publicly accessible, de-identified transcriptomic and clinical data. The Medical Ethics Committee of Quanzhou Medical College determined that this secondary analysis of publicly available, de-identified data did not require ethical approval; no reference number was issued. No additional informed consent was required. Ethics approvals and consent procedures for the original data collection are described in the corresponding source publications and repository records. Reporting followed REMARK and STROBE principles [5,6].

The primary estimand was the association between the fixed 15-gene cohort-relative score and source-study-defined pCR. We estimated cohort-specific ORs for pCR per one-standard-deviation increase in the primary cohort-relative score and summarized these associations across four accession-defined cohorts. Because GSE25055 and GSE25065 arose from the same Hatzis/MD Anderson parent study series, we also summarized the evidence at the level of three principal study sources. The study evaluates cross-cohort reproducibility of an association; clinical deployment and treatment-effect prediction were outside the estimand.

The primary clinical analysis included pretreatment tumors with strict TNBC annotation and a binary pCR versus residual-disease outcome. The retained accession-defined cohorts were GSE25055 and GSE25065 from the Hatzis taxane-anthracycline series [7], GSE32646 [8], and GSE163882 [9]. Treatment and pathologic-response definitions were retained as specified by the source studies rather than retrospectively harmonized. GSE32646 defined pCR as no residual invasive cancer in both breast and axilla. GSE163882 used the pathology-report definition from each contributing institution. For GSE25055/GSE25065, the source binary pCR label was retained; breast/node/DCIS details not separately specified in the accessible main report were not inferred. Cohort characteristics are summarized in Table 1.

**Table 1:** Characteristics of the four accession-defined strict-TNBC cohorts used in the primary pCR analysis.

Cohort	Parent study/source	Platform and pretreatment specimen	Neoadjuvant treatment	Source pCR definition retained	n/pCR
GSE25055	Hatzis et al. 2011; GSE25066 discovery cohort	GPL96 Affymetrix U133A; pretreatment FNA or core biopsy	Sequential taxane- and anthracycline-based chemotherapy	Parent report: no invasive/metastatic breast cancer after NAC; source pCR/RD retained	112/37
GSE25065	Hatzis et al. 2011; GSE25066 validation cohort	GPL96 Affymetrix U133A; pretreatment FNA or core biopsy	Sequential taxane- and anthracycline-based chemotherapy	Parent report: no invasive/metastatic breast cancer after NAC; source pCR/RD retained	58/20
GSE32646	Miyake et al. 2012	GPL570 Affymetrix U133 Plus 2.0; pretreatment vacuum-assisted core biopsy	Paclitaxel followed by FEC	No residual invasive cancer in both breast and axilla	26/10
GSE163882	Chen et al. 2021 multicenter cohort	Illumina NextSeq 500; FFPE pretreatment biopsy; processed TPM	Anthracycline-taxane-based chemotherapy; regimen determined by treating institutions	pCR/RD as defined in the pathology report by the tissue block’s institution of origin	89/37

Treatment and pCR definitions are reported as specified by the source studies. For GSE25055/GSE25065, the parent report described pCR as no invasive or metastatic breast cancer after neoadjuvant chemotherapy; nodal/DCIS details were not further harmonized. GSE25055 and GSE25065 are separate accessions from the same parent study and were combined as one source only in the source-structure sensitivity analysis.

GSE319641 supplied paired baseline and day 1 cycle 2 (D1C2) NeoTRIP profiles [10,11], and the 10-TNBC subset of GSE176078 supplied the single-cell source data used here [12]. GSE194040 was retained as a contextual HR-negative/HER2-negative I-SPY2 resource [13]. The patient-level I-SPY2 analysis included treatment-arm-adjusted, arm+MammaPrint, and score-by-arm interaction models. Because I-SPY2 differs from the primary strict-TNBC cohorts in receptor eligibility and multi-arm treatment structure, it remained outside the primary meta-analysis.

### Patient-overlap and duplicate-aliquot assessment

Before modeling, patient identifiers were harmonized using deterministic source-ID rules. In the GSE20194/GSE25055 audit, matching was based on patient identifiers; receptor annotation, pCR concordance, and expression similarity were secondary checks rather than matching criteria. Direct screening of public GSE20194 metadata for ER-negative, PR-negative, HER2-negative tumors with a non-missing binary pCR/residual-disease outcome identified 71 records. Three documented R1 repeat records (M233R1, M375R1, and M402R1) were collapsed to their underlying patients, leaving 68 unique patients. Fifty-eight patient identifiers were present in GSE25055; 57 satisfied strict-TNBC criteria in both accessions, while M180 had concordant pCR but discordant PR annotation and was not included in the strict-to-strict duplicate set. Ten GSE20194 patients had no matching GSE25055 identifier. Because participant reuse was extensive, GSE20194 was excluded as an independent primary cohort; the unmatched residual subset was not retained as a post hoc cohort defined by overlap status.

In GSE163882, BA00095 and BA00096 mapped to patient S10-7089 [9]. Their expression-derived composite values were collapsed at patient level before final cohort re-standardization. All clinical analyses used one row per patient.

To illustrate the effect of accession-level duplicate weighting, the 57 confirmed strict-to-strict duplicated patients were analyzed as a duplicate-only GSE20194 subset and that accession effect was naively added to the four primary cohort effects. This counterfactual was descriptive only and did not add independent participants.

### Pathway definition and score construction

The primary score was anchored to the 16-gene MitoCarta3.0 Chaperones pathway [3]. The minimum 80% common-platform coverage rule was applied across GPL96, GPL570, and the I-SPY2 expression platform. Fifteen genes were reliably measurable across those three platforms: DNAJA3, FKBP10, HSPD1, HSPE1, MAIP1, NRDC, PHB1, PHB2, TIMM10, TIMM10B, TIMM13, TIMM8A, TIMM8B, TIMM9, and TRAP1. PHB was mapped to PHB1; the aliases C2orf47 to MAIP1 and NRD1 to NRDC were used where required. DNLZ was excluded because GPL96 lacked a reliable probe. GSE163882 was incorporated after confirming that all 15 target genes were present in its processed RNA-seq matrix. The 15-gene set, equal positive weights, expected direction (higher score associated with lower pCR probability), platform-coverage rule, and no-cutpoint rule defined the primary score specification used in this study. Project records describe this specification as fixed before the present score-specific pCR analysis, after related pCR analyses using a different mitochondrial gene set in overlapping public cohorts. This was a retrospective specification, not a public preregistration or a fully outcome-blinded development process. The analytical history and subsequent analysis changes are documented in S1 File.

In the microarray cohorts, probes were mapped to unambiguous current gene symbols and multiple retained probes for a gene were summarized by their within-sample median. For GSE163882, TPM values were transformed as log2(TPM + 1), and rows with the same gene symbol after alias harmonization were summarized by their within-sample maximum. Each of the 15 target genes was z-standardized using all samples in that accession’s public processed expression matrix (222 profiles for GSE163882). For GSE163882, aliquot-level composites for BA00095 and BA00096 were averaged for patient S10-7089 before the final composite was re-standardized across the 89 retained patients. Formally, for target gene g and sample i in accession a, z_gia=(x_gia-mean_ga)/SD_ga; the equal-weight composite was m_ia=(1/15) sum_g z_gia; and the reported analysis score was S_ic=(m_ia-mean_c[m])/SD_c[m] within analysis cohort c after restriction to retained patients and patient-level aliquot handling. Changing the accession reference changes mean_ga/SD_ga, and changing the analysis cohort changes mean_c[m]/SD_c[m]; the same biological sample can therefore receive a different numerical score under a different reference distribution. The primary score is cohort-relative rather than a fixed single-sample clinical assay.

### Primary clinical models and meta-analysis

Cohort-specific logistic regression estimated the OR for pCR per one within-cohort SD increase in the primary score. The raw-score model was primary. Exploratory models added a composite proliferation score and then proliferation, ESTIMATE immune score, and ESTIMATE stromal score [14,15]. A proliferation-only model provided directional context. A post hoc within-cohort residual score was obtained by regressing the primary score on proliferation, immune, and stromal scores separately within each cohort and then z-standardizing the residual. Because nuisance coefficients and residual scale were estimated in the same cohort used for the outcome model, this residual analysis was treated as biological decomposition rather than external validation.

Cohort-specific log-ORs were pooled using random-effects meta-analysis with restricted maximum-likelihood (REML) estimation of between-study variance and modified Hartung-Knapp (mKH) inference [16,17]. The tau^2 = 0 boundary was compared with the numerical REML optimum. With only three to four analysis units, I^2 was treated as descriptive. As an estimator sensitivity, tau^2 was also estimated with the Paule-Mandel method while retaining the same mKH inference.

The primary meta-analysis used the four accession-defined cohorts. In a source-structure sensitivity analysis, GSE25055 and GSE25065 retained separate accession intercepts and their accession-specific score scaling but contributed one common score slope for the Hatzis/MD Anderson source. That source effect was pooled with GSE32646 and GSE163882 to give three principal study-source effects.

No model-specific attrition occurred in the retained score, proliferation, immune, stromal, residual, leave-one-cohort-out (LOCO), or rank-score analyses: GSE25055 contributed 112 patients/37 pCR events, GSE25065 58/20, GSE32646 26/10, and GSE163882 89/37, for a total of 285/104.

### Fixed single-sample rank-score sensitivity

As an implementation sensitivity, we used one outcome-independent single-sample rank score with the same 15 genes and positive directions. The implementation used a 12,151-gene background universe measurable across GPL96, GPL570, and the GSE163882 RNA-seq matrix, together with defined alias and multi-probe handling. Within each sample, genes were ranked across this fixed universe and

RankScore = [mean rank of the 15 target genes – (15 + 1)/2]/ (G-15), with G = 12,151. Ranks are ascending within sample across the fixed universe, and tied values receive their average rank.

The primary effect unit was an OR for pCR per 0.10-unit increase in RankScore; ORs per one within-cohort SD were reported secondarily for scale comparison. The same 285 patients were analyzed. A single RankScore definition was applied uniformly across the four primary cohorts. This sensitivity reduces dependence on cross-patient gene-level z-standardization while retaining the upstream processed expression matrices.

### Proliferation, immune, and tissue-composition analyses

Proliferation was represented by *MKI67*, Hallmark E2F targets, Hallmark G2M checkpoint, and a standardized composite [14]. ESTIMATE immune and stromal scores were used as expression-derived tissue-composition proxies [15]. CIBERSORTx LM22 relative fractions were estimated with 1,000 permutations using platform-appropriate preprocessing [18], and MCP-counter supplied a broad-lineage analysis [19]. Spearman correlations and cell-fraction-adjusted logistic models were meta-analyzed across cohorts. Benjamini-Hochberg false-discovery-rate correction [20] was applied within the prespecified cell-type families. These analyses provided exploratory biological context.

### Leave-one-cohort-out and leave-one-source-out nuisance-relation diagnostics

For four-fold leave-one-cohort-out (LOCO) residualization, nuisance slopes relating the primary score to standardized proliferation, immune, and stromal variables were estimated in the other cohorts with training-cohort intercepts. The held-out residual was expressed in units of the training-set residual SD, and a residual-only pCR model was then fitted in the held-out cohort. This residual-only model estimates whether the nuisance relationship learned in other cohorts transports to the held-out cohort; it estimates a different parameter from the direct multivariable model that retains the raw score and background covariates.

For each held-out cohort we additionally recorded the training residual SD, the held-out residual SD expressed in training-SD units, and Pearson correlations between the held-out residual and proliferation, immune, and stromal variables. A source-level diagnostic held GSE25055 and GSE25065 out together; when both accessions were evaluated together, the pCR model used accession-specific intercepts and one shared residual slope. Source-level effects were pooled across the three principal sources using the same REML+mKH procedure.

### NeoTRIP paired longitudinal analysis

The NeoTRIP analysis used 150 patients with paired baseline and D1C2 profiles from GSE319641 [10,11]. The raw 15-gene score was calculated from the provider-supplied linear-scale matrix: each target gene was z-standardized across all 401 supplied profiles using population SD, the 15 z scores were averaged, and the composite was again standardized across all 401 profiles.

Within-patient raw-score changes were tested with paired t and Wilcoxon signed-rank tests. The E2F, G2M, ESTIMATE, proliferation-composite, residual, and joint-change calculations used the same processed inputs. These decompositions were interpreted as exploratory context.

### Single-cell source analysis

The analysis used the 10-TNBC subset of GSE176078 [12], represented by 42,512 cells in the processed single-cell data. Source-provided patient identifiers (`orig.ident`) and cell-type annotations were retained; `Cancer Epithelial` was used as the malignant epithelial compartment. Counts were aggregated by patient and major cell type, converted to counts per 10,000, transformed with log1p, and averaged across the 15 target genes. Patient-level pseudobulk was the inferential unit.

For the descriptive cell-type display, patient-cell-type groups with at least 20 cells were retained and pseudobulk scores were centered within patient across eligible major cell types. For the paired malignant-versus-noncancer comparison, a patient required one `Cancer Epithelial` row and at least one non-cancer row with positive pooled counts. Eight of 10 TNBC patients met this rule; CID3946 and CID44041 lacked a `Cancer Epithelial` row. All malignant epithelial counts and all non-cancer counts were pooled separately within each eligible patient before the same counts-per-10,000, log1p, and 15-gene averaging steps. Paired scores were compared with the exact two-sided Wilcoxon signed-rank test. A cycling-state sensitivity was evaluated separately; the main comparison was malignant epithelium versus pooled non-cancer cells.

### Statistical software and reproducibility

Core analyses were performed in Python 3.13.5 using pandas 2.2.3, NumPy 2.3.5, SciPy 1.17.0, and statsmodels 0.14.6; figures were generated with matplotlib 3.10.8 and Pillow 12.2.0. S1 File provides the derived data and scripts used to reproduce the cohort models, meta-analyses, LOCO procedure, and score construction.

S1 File provides executable workflows for the core clinical models, RankScore, source-level diagnostics, patient-level I-SPY2 models, and reduced-input single-cell analysis. For NeoTRIP, it provides derived sample-level data, numerical outputs, historical analysis code, and archived verification records; the historical generator requires the original project inputs and path configuration. All tests were two-sided. Additional supplementary methods, tables, and figures are provided in S1 Appendix.

### Use of generative artificial intelligence

OpenAI ChatGPT was used for language editing, code review, and consistency checking. The authors are responsible for all data, analyses, results, and manuscript content.

## Results

### Patient-level matching identified extensive reuse between GSE20194 and GSE25055

Screening of public GSE20194 metadata identified 71 strict-TNBC records. Three repeat records represented the same underlying patients, leaving 68 unique patients. Fifty-eight of the 68 patient identifiers were present in GSE25055; 57 patients satisfied strict-TNBC criteria in both accessions, one identifier-matched patient had discordant PR annotation, and 10 had no matching GSE25055 identifier. GSE20194 was excluded as an independent cohort, so the primary analysis used the four non-overlapping accession-defined cohorts (Fig 1).

**Fig 1:**
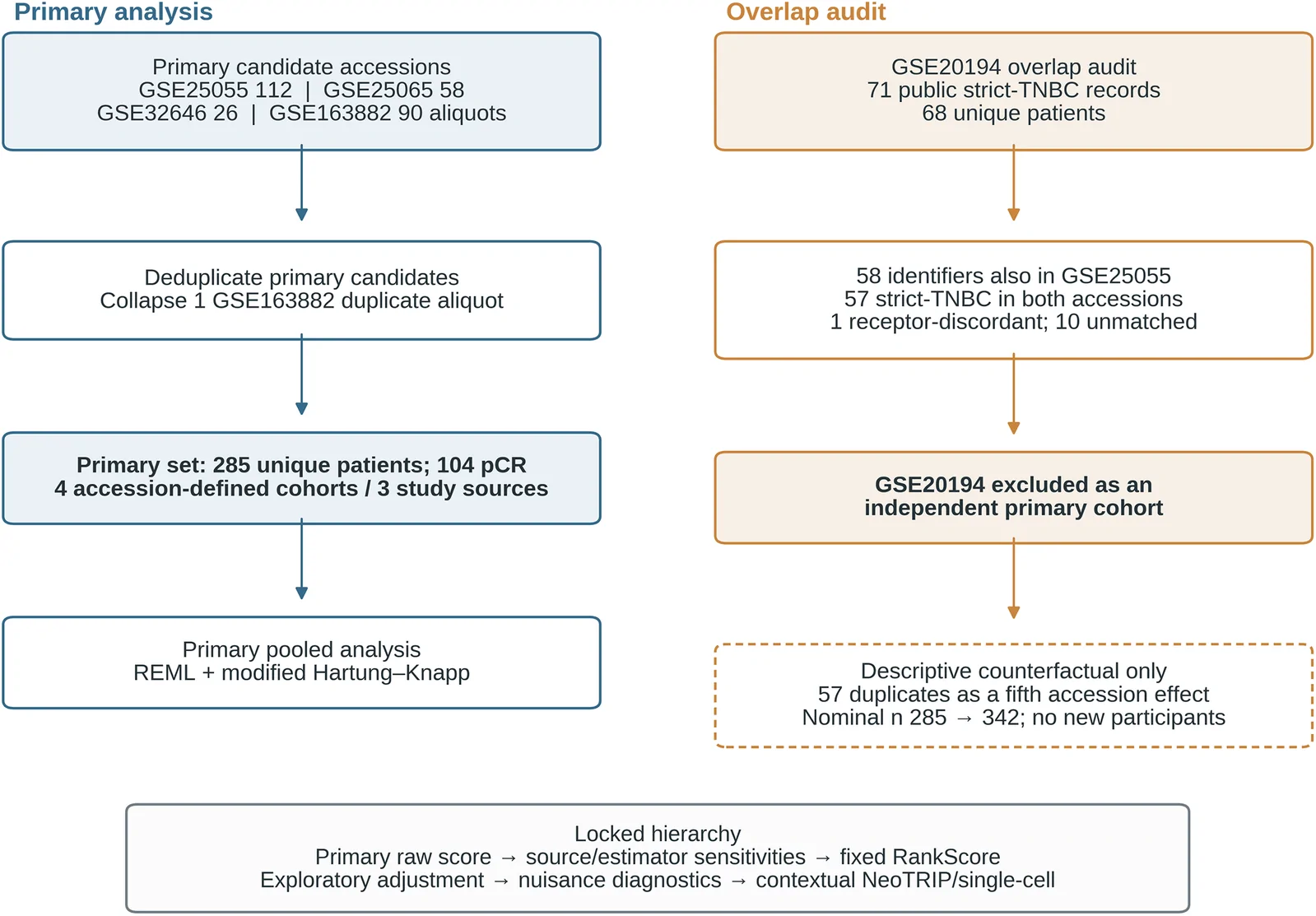
Study design, patient overlap, and analysis hierarchy. The primary pCR analysis includes four accession-defined strict-TNBC cohorts from three principal study sources (n = 285; 104 pCR). Screening of GSE20194 identified 71 strict-TNBC records, 68 unique patients after three R1-repeat collapses, 58 patient identifiers present in GSE25055, 57 strict-TNBC patients in both accessions, one receptor-discordant identifier match, and 10 unmatched GSE20194 patients. GSE20194 was excluded as an independent cohort. The analysis hierarchy distinguishes the primary raw-score association from source-structure and score-implementation sensitivities, exploratory biological-context models, nuisance-relation diagnostics, and contextual longitudinal/single-cell analyses.

After collapse of the duplicated GSE163882 aliquot, the primary analysis comprised 285 unique patients from four accession-defined cohorts, including 104 with source-study-defined pCR (Table 1). GSE25055 and GSE25065 contributed 170 of these patients but arose from the same parent Hatzis/MD Anderson study source.

Among the 57 confirmed strict-to-strict duplicated patients, the GSE20194 score was not associated with pCR (OR per SD, 1.028; 95% CI, 0.603–1.752; P = 0.919). Naively treating this duplicate-only subset as a fifth accession effect increased the nominal sample size from 285 to 342 and the nominal pCR count from 104 to 126, while the pooled estimate changed only slightly (OR 0.971; 95% CI, 0.705–1.337; P = 0.808). The added records therefore increased nominal information by double-counting patients rather than by adding independent participants.

### The primary association was imprecise across four cohorts and three principal study sources

Across the four accession-defined strict-TNBC cohorts, the primary REML+mKH pooled association was OR 0.958 per one within-cohort score SD (95% CI, 0.639–1.437; P = 0.760; tau^2 = 0; I^2 = 0%). Paule-Mandel+mKH gave the same four-cohort estimate and interval. With four cohorts, I^2 was interpreted descriptively (Fig 2).

**Fig 2:**
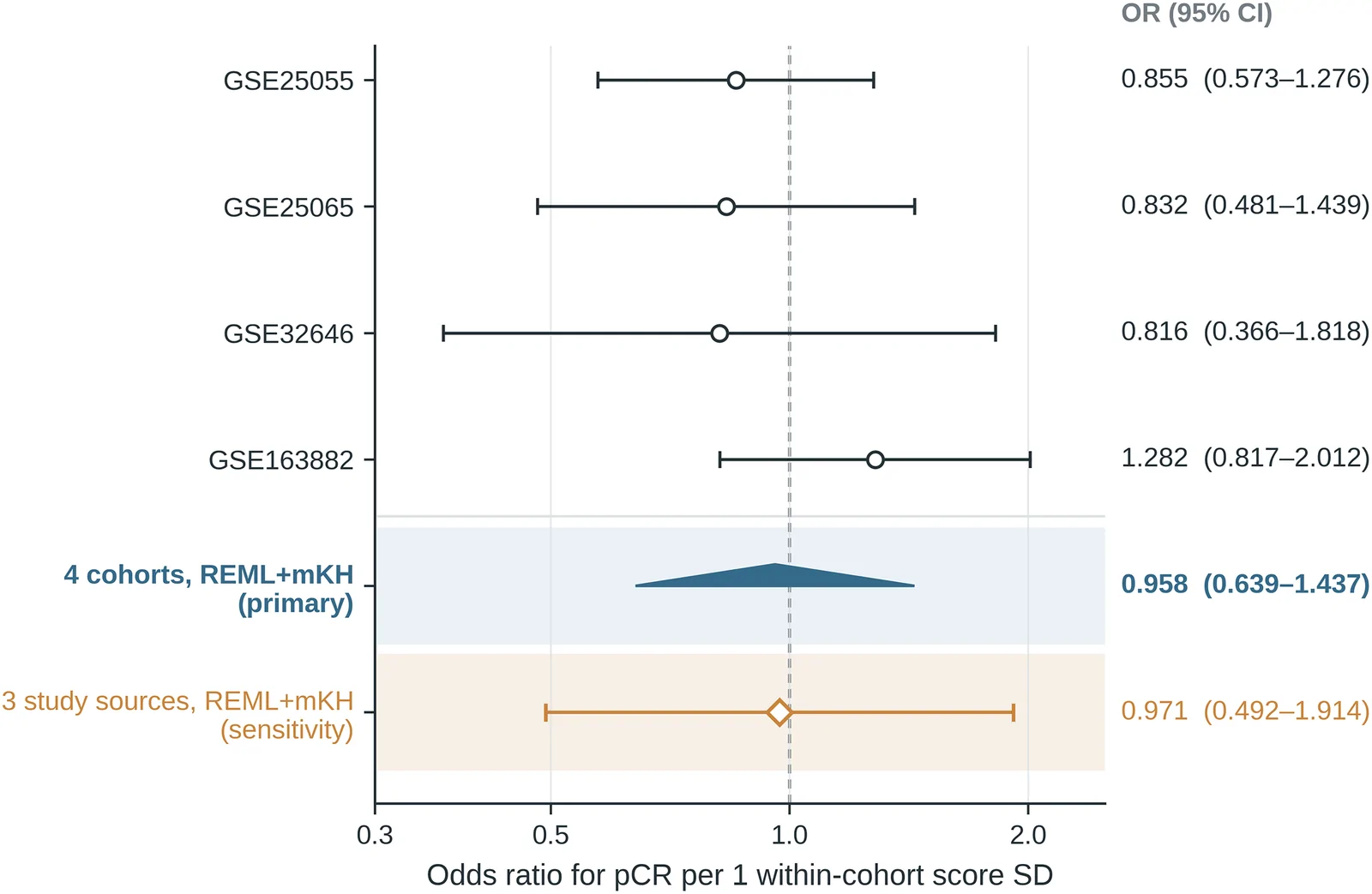
Primary raw-score associations with pCR across the four accession-defined cohorts. Cohort-specific points are ORs per one within-cohort SD increase in the primary score. The primary pooled diamond is the four-cohort REML random-effects estimate with modified Hartung-Knapp inference. A separately labeled pooled row summarizes the three principal study sources as a sensitivity analysis in which GSE25055 and GSE25065 contribute one shared source slope. The dashed vertical line marks OR=1.

When GSE25055 and GSE25065 were summarized as one Hatzis/MD Anderson study source, the three-source REML+mKH estimate was OR 0.971 (95% CI, 0.492–1.914; P = 0.868; tau^2 = 0.0201; I^2 = 13.7%). Paule-Mandel+mKH yielded OR 0.965 (95% CI, 0.530–1.757; P = 0.820; tau^2 = 0.00717). The source-level confidence intervals were substantially wider than the four-accession interval and remained compatible with associations in either direction (Table 2; Fig 2).

**Table 2:** Primary, sensitivity, exploratory, and nuisance-relation estimates for pCR.

Analysis	Effect unit	n/pCR	OR (95% CI)	P	Heterogeneity/role
Primary raw score, four cohorts (primary; REML+mKH)	1 within-cohort raw-score SD	285/104	0.958 (0.639-1.437)	0.760	tau^2=0; I^2=0%
Primary raw score, three sources (REML+mKH)	accession-scaled score; shared source slope for the two Hatzis accessions	285/104	0.971 (0.492-1.914)	0.868	tau^2=0.0201; I^2=13.7%; source sensitivity
Primary raw score, three sources (PM + mKH)	same as preceding row	285/104	0.965 (0.530-1.757)	0.820	tau^2 = 0.00717; estimator sensitivity
Fixed RankScore, four cohorts	+0.10 RankScore	285/104	0.613 (0.148-2.542)	0.354	tau^2=0; I^2=0%; implementation sensitivity
Score + proliferation	1 within-cohort raw-score SD, adjusted	285/104	0.553 (0.317-0.967)	0.043	exploratory
Score + proliferation + immune + stromal	1 within-cohort raw-score SD, adjusted	285/104	0.473 (0.252-0.888)	0.032	exploratory
Same-cohort residual	1 within-cohort residual SD	285/104	0.633 (0.413-0.968)	0.042	post hoc decomposition
Four-fold LOCO residual	1 fold-specific training residual SD	285/104	0.790 (0.439-1.420)	0.290	tau^2=0.0695; I^2=53.7%; nuisance-relation diagnostic
Leave-one-study-source-out residual	1 source-fold training residual SD	285/104	0.851 (0.345-2.095)	0.521	tau^2=0.0772; I^2=64.1%; source-level diagnostic

The direct adjusted, same-cohort residual, and LOCO residual rows estimate different conditional or transformed associations and use different residual scales.

In the fixed single-sample rank sensitivity, all four cohort-specific point estimates were below 1 but were imprecise. The four-cohort pooled estimate was OR 0.613 per 0.10-unit RankScore increase (95% CI, 0.148–2.542; P = 0.354; tau^2 = 0; I^2 = 0%), and the three-source estimate was OR 0.612 (95% CI, 0.090–4.170; P = 0.386). On a one-within-cohort-SD scale, the four-cohort rank estimate was OR 0.868 (95% CI, 0.585–1.288; P = 0.337). Within-cohort Pearson correlations between RankScore and the primary score ranged from 0.630 to 0.953. Both fixed implementations therefore gave imprecise source-level inference, with different point-estimate magnitudes.

### Proliferation and tissue context were associated with the primary score, but adjusted and residual analyses answer different questions

The primary score correlated positively with the proliferation composite in every primary cohort (cohort Spearman rho = 0.441–0.634; pooled rho = 0.544; 95% CI, 0.375–0.679; P = 0.003). Immune and stromal correlations varied across cohorts. In same-cohort exploratory outcome models, adjustment for proliferation gave an OR for the primary score of 0.553 (95% CI, 0.317–0.967; P = 0.043), and adjustment for proliferation, immune, and stromal scores gave OR 0.473 (95% CI, 0.252–0.888; P = 0.032). The post hoc within-cohort residual analysis gave OR 0.633 per within-cohort residual SD (95% CI, 0.413–0.968; P = 0.042). These estimates are exploratory decompositions. Proliferation alone had OR 1.645 (95% CI, 0.979–2.764; P = 0.055).

The four-fold residual-only LOCO diagnostic gave a pooled OR of 0.790 per fold-specific training residual SD (95% CI, 0.439–1.420; P = 0.290; tau^2 = 0.0695; I^2 = 53.7%). Training residual SDs ranged from 0.777 to 0.816, whereas held-out residual SDs ranged from 0.919 to 1.226 in training-SD units. Held-out residuals were not uniformly orthogonal to the background variables: in GSE163882, Pearson correlations with proliferation, immune, and stromal scores were 0.547, 0.470, and 0.625, respectively. The nuisance relationship estimated in other cohorts therefore did not transfer uniformly to every held-out cohort (Fig 3).

**Fig 3:**
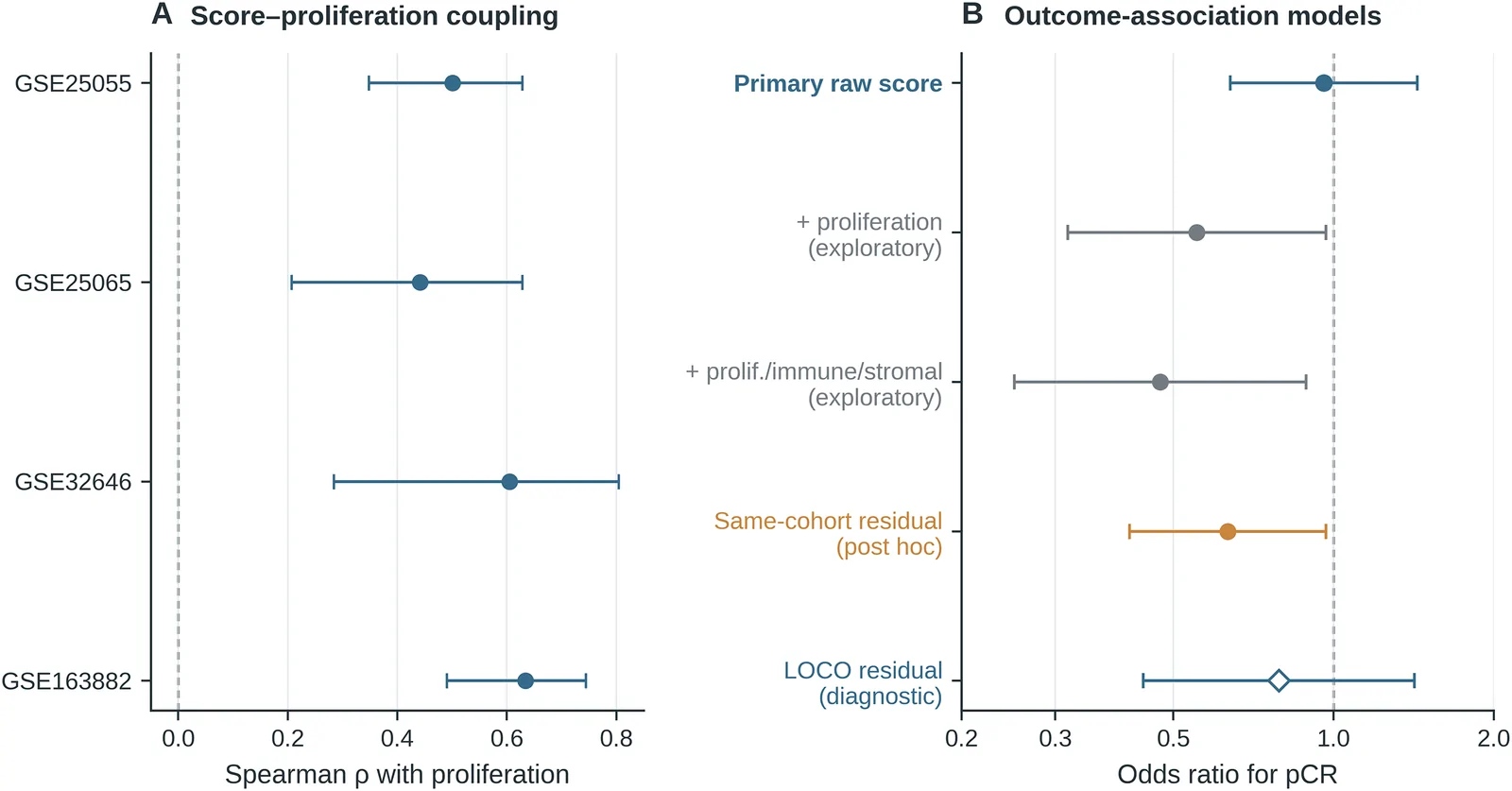
Proliferation coupling, exploratory decomposition, and external nuisance-relation diagnostics. (a) Cohort-specific Spearman correlations between the primary score and the proliferation composite. (b) Association estimates from the primary raw-score model, direct exploratory adjusted models, same-cohort residual model, and residual-only LOCO diagnostic. Raw/adjusted score effects are per one within-cohort raw-score SD; same-cohort residual effects are per one within-cohort residual SD; LOCO effects are per fold-specific training residual SD.

When GSE25055 and GSE25065 were held out together as one study source, the three-source leave-out diagnostic remained imprecise (pooled OR 0.851 per source-fold training residual SD; 95% CI, 0.345–2.095; P = 0.521; tau^2 = 0.0772; I^2 = 64.1%). The source-level result was consistent with limited transport of the nuisance relationship across the three independent study sources.

### No stable immune-cell lineage association survived multiplicity correction

Across the immune analyses, no LM22 cell-type correlation with the score survived false-discovery-rate correction, and no cell fraction remained associated with pCR after adjustment for the score and multiplicity correction. MCP-counter likewise yielded no false-discovery-rate-significant score-lineage correlation. These analyses identified no reproducible immune-lineage correlate of the score after multiplicity correction.

### NeoTRIP showed an early raw-score decrease

Among 150 paired NeoTRIP patients, the raw score decreased from a mean of 0.384 at baseline to −0.511 at D1C2, for a mean within-patient change of −0.894 all-401-standardized score units (95% CI, −1.050 to −0.738; paired t-test P = 6.70 x 10^-22; Fig 4).

**Fig 4:**
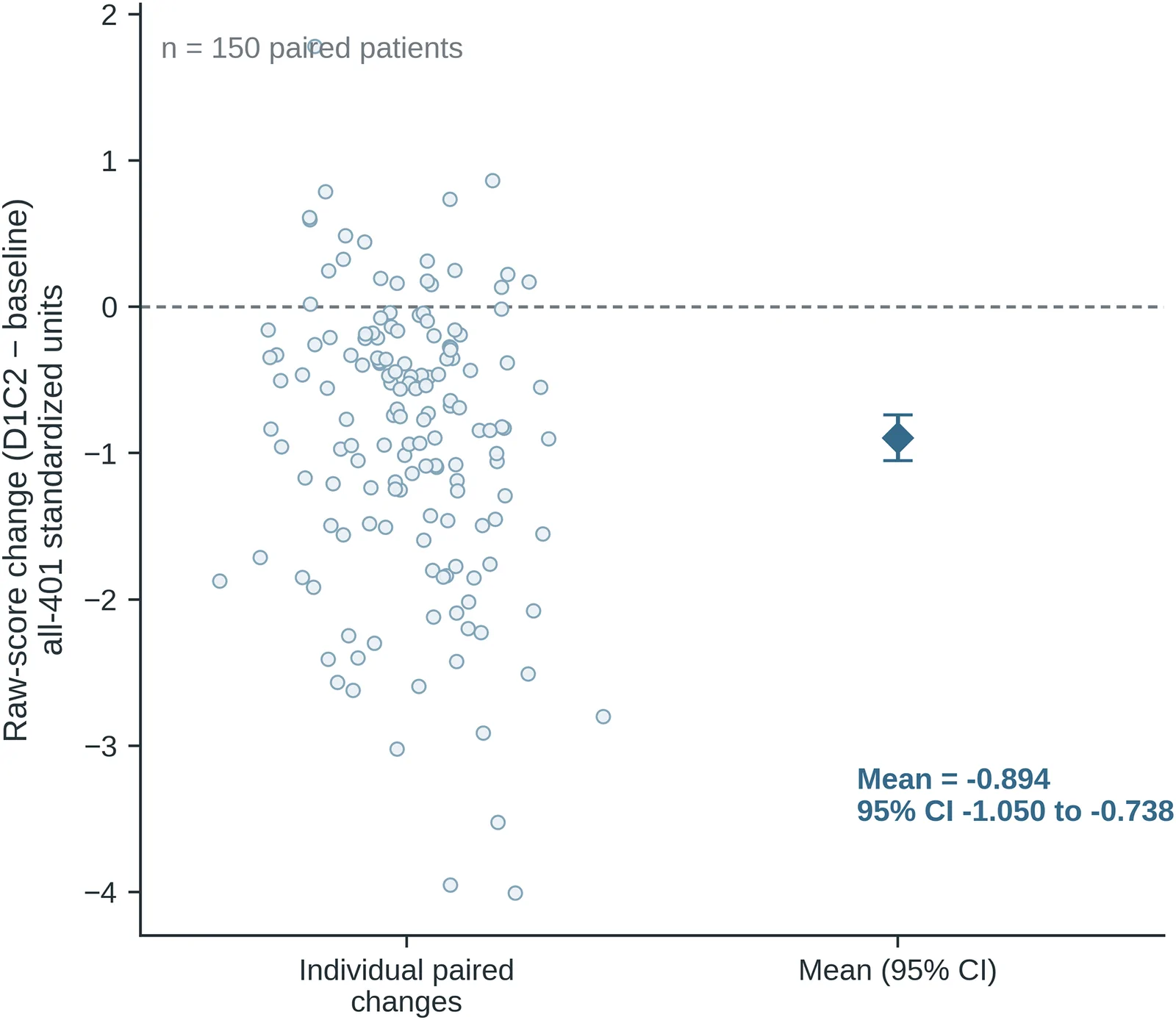
Early raw-score change in paired NeoTRIP samples. The raw-score implementation uses the provider-supplied linear-scale matrix and all 401 profiles as the gene/composite standardization reference. Among the 150 paired patients, the score decreased from baseline to D1C2 by a mean of 0.894 standardized units.

E2F/G2M/ESTIMATE-derived proliferation, immune, stromal, residual, and joint-change calculations were interpreted as exploratory context. The raw-score decrease is a bulk-tissue longitudinal observation, and the decomposition was interpreted as biological context rather than a tumor-cell-intrinsic mitochondrial mechanism.

### Single-cell pseudobulk showed malignant-epithelial enrichment relative to a pooled non-cancer compartment

The single-cell source data contained 42,512 cells from the 10-TNBC subset of GSE176078. Source-provided major-cell-type labels were retained, with `Cancer Epithelial` used as the malignant epithelial compartment. Eight patients had one malignant epithelial row plus non-cancer rows with positive pooled counts; CID3946 and CID44041 did not meet this criterion. The pooled non-cancer score was calculated by summing all non-cancer counts within patient before transformation and scoring. Malignant epithelium had a higher score in all eight paired patients; the median paired difference was 0.260 and the exact two-sided Wilcoxon signed-rank P value was 0.0078 (Fig 5). This patient-level analysis supports relative malignant-epithelial enrichment.

**Fig 5:**
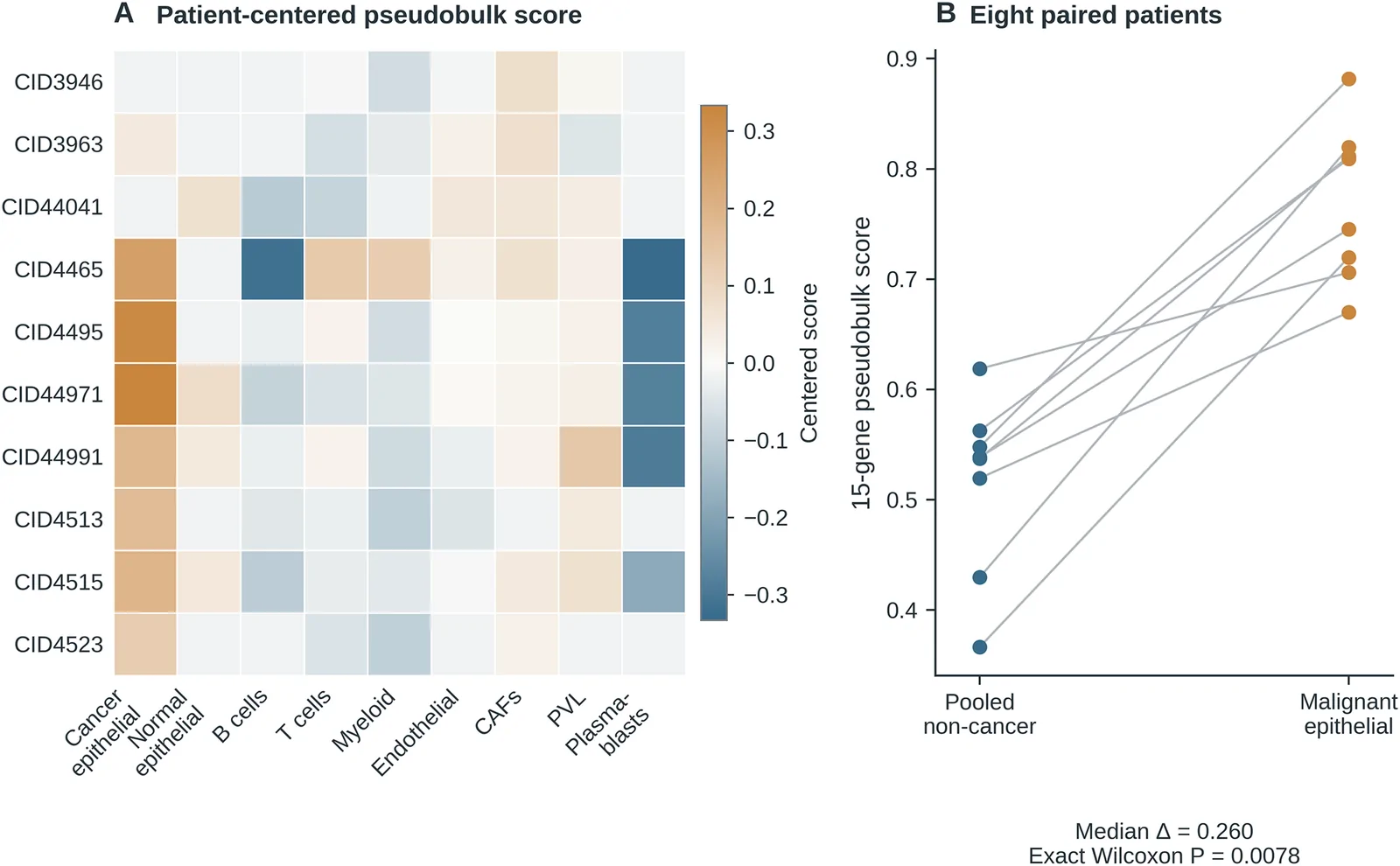
Patient-level single-cell localization analysis. (a) Patient-by-major-cell-type pseudobulk 15-gene scores using source-provided `celltype_major` labels; `Cancer Epithelial` is the malignant epithelial label used here. Patient-cell-type groups of at least 20 cells are retained for display and scores are centered within patient across eligible major cell types; gray cells indicate unavailable/not-displayed combinations. (b) Paired malignant epithelial versus pooled-count non-cancer compartment scores for the eight eligible patients. All non-cancer counts were pooled within patient before counts-per-10,000 normalization, log1p transformation, and 15-gene averaging. Malignant epithelium was higher in all eight pairs (median difference 0.260; exact two-sided Wilcoxon P = 0.0078).

### Contextual I-SPY2 analysis

The patient-level GSE194040 analysis included treatment-arm-adjusted, arm+MammaPrint, and score-by-arm interaction models. I-SPY2 was treated as contextual because its HR-negative/HER2-negative eligibility and multi-arm treatment structure differ from the strict-TNBC primary cohorts.

## Discussion

Across four accession-defined strict-TNBC cohorts from three principal study sources, the primary 15-gene cohort-relative score did not show a reproducible association with pCR. The primary four-cohort estimate was close to the null, the three-source estimate was similarly centered but less precise, and the fixed single-sample RankScore analysis was also imprecise. Together, the two fixed implementations did not identify a stable cross-cohort association.

The overlap analysis shows why patient provenance matters in public-cohort biomarker studies. Fifty-seven strict-TNBC patients were represented in both GSE20194 and GSE25055. Treating those patients as an additional accession effect increased nominal n from 285 to 342 without adding independent participants. The pooled point estimate changed little here, but duplicate weighting can alter precision and effect estimates. Cross-cohort analyses should establish participant independence from source identifiers and provenance rather than accession labels alone.

The score is reference-dependent. Each target gene was standardized against all samples in its accession-level processed matrix before the 15-gene composite was formed, and the composite was then re-standardized within the final analysis cohort. The implementation therefore measures position relative to study-specific expression distributions rather than a fixed single-sample scale. The fixed RankScore sensitivity reduced one aspect of cross-patient reference dependence. On a common one-within-cohort-SD scale, the primary-score estimate was OR 0.958 and the RankScore estimate was OR 0.868; both confidence intervals crossed 1. The two fixed implementations therefore produced similarly imprecise source-level inference.

Direct adjustment and residual-only LOCO answer different questions. The LOCO residuals were scaled to training-set residual SDs, and nuisance relationships learned outside the held-out cohort did not remain orthogonal in every cohort. The LOCO analysis showed that the empirical relationship between the score and broad background programs varied across study contexts.

The biological-context analyses add localization and covariate information. The score correlated with proliferation across the four primary cohorts, while immune and stromal relationships varied. Single-cell pseudobulk showed relative malignant-epithelial enrichment in eight paired patients. In NeoTRIP, the bulk-tissue score decreased early during therapy, while E2F/G2M/ESTIMATE-derived analyses provided proliferation and tissue-composition context. These results characterize the biological context of the score rather than a tumor-cell-intrinsic mitochondrial mechanism.

Precision is limited by the small number of independent study sources. GSE25055 and GSE25065 are separate accessions from the same Hatzis/MD Anderson parent series, leaving three principal sources. The primary-score three-source confidence interval was approximately 0.49–1.91, and the fixed RankScore source-level interval was wider. With so few units, I^2 and tau^2 estimates are themselves imprecise; the effect estimates and confidence intervals are more informative than heterogeneity point estimates alone.

I-SPY2 provides contextual information from a different receptor-defined, multi-arm treatment setting. The patient-level analysis included the adjusted and interaction models. Its HR-negative/HER2-negative eligibility and treatment structure differ from the strict-TNBC primary cohorts, so I-SPY2 was not included in the primary meta-analysis. Treatment-effect prediction requires a prespecified treatment-selection framework and reproducible score-by-treatment interaction evidence.

Future validation should use independent study populations, a fixed single-sample scoring implementation or external reference distribution, prospectively specified preprocessing and analysis rules, and more independent study sources. Treatment-selection studies should prespecify score-by-treatment interaction analyses.

Prior examination of related gene sets and pCR outcomes in overlapping cohorts leaves potential analytical flexibility despite the fixed specification of the present score. The study remains limited by its retrospective design, heterogeneity among public cohorts in treatment, specimen type, pathology definitions, expression platform, and available covariates, and only three principal study sources. pCR followed the source-study definitions. The primary analysis contains 285 patients, including one cohort with 26 patients and 10 pCR events. ESTIMATE and digital cytometry are expression-derived proxies rather than direct histology, and RankScore still depends on upstream processed expression matrices. NeoTRIP lacked linked patient-level clinical outcomes in the available data used for this analysis. The single-cell localization analysis included 10 tumors, with eight informative paired compartments, and used source-provided cell-type labels. These features limit clinical deployment, treatment-selection, and causal-mechanism inference.

## Conclusions

Across four accession-defined cohorts from three principal study sources, the evaluated cohort-relative 15-gene score implementations did not show a reproducible association with source-study-defined pCR. Source-level estimates remained imprecise. Robust cross-cohort biomarker evaluation requires patient-level deduplication, explicit study-source accounting, transparent reference distributions, reproducible score construction, and clear separation of association, biological decomposition, and treatment-effect prediction.

## Supporting information

S1 Appendix Supplementary methods, cohort and model-accounting tables, patient-overlap analysis, score-implementation sensitivity, three-source and Paule-Mandel meta-analysis sensitivity, LOCO held-out diagnostics, immune analyses, and contextual NeoTRIP, single-cell, and I-SPY2 analyses. (PDF)

S1 File Derived data, public-input manifest, executable core and contextual-analysis workflows, result tables, figure source data, and verification outputs. (ZIP)

## References

[1] Spring LM, Fell G, Arfe A, Sharma C, Greenup R, Reynolds KL, et al. Pathologic complete response after neoadjuvant chemotherapy and impact on breast cancer recurrence and survival: A comprehensive meta-analysis. Clin Cancer Res. 2020;26(12):2838–48. doi: 10.1158/1078-0432.CCR-19-3492 32046998 PMC7299787

[2] Shi L, Campbell G, Jones WD, Campagne F, Wen Z, Walker SJ, et al. The MicroArray Quality Control (MAQC)-II study of common practices for the development and validation of microarray-based predictive models. Nat Biotechnol. 2010;28(8):827–38. doi: 10.1038/nbt.1665 20676074 PMC3315840

[3] Rath S, Sharma R, Gupta R, Ast T, Chan C, Durham TJ, et al. MitoCarta3.0: an updated mitochondrial proteome now with sub-organelle localization and pathway annotations. Nucleic Acids Res. 2021;49(D1):D1541–7. doi: 10.1093/nar/gkaa1011 33174596 PMC7778944

[4] Baek ML, Lee J, Pendleton KE, Berner MJ, Goff EB, Tan L, et al. Mitochondrial structure and function adaptation in residual triple negative breast cancer cells surviving chemotherapy treatment. Oncogene. 2023;42(14):1117–31. doi: 10.1038/s41388-023-02596-8 36813854 PMC10069007

[5] McShane LM, Altman DG, Sauerbrei W, Taube SE, Gion M, Clark GM, et al. REporting recommendations for tumour MARKer prognostic studies (REMARK). Br J Cancer. 2005;93(4):387–91. doi: 10.1038/sj.bjc.6602678 16106245 PMC2361579

[6] von Elm E, Altman DG, Egger M, Pocock SJ, Gøtzsche PC, Vandenbroucke JP, et al. The strengthening the reporting of observational studies in epidemiology (STROBE) statement: Guidelines for reporting observational studies. PLoS Med. 2007;4(10):e296. doi: 10.1371/journal.pmed.0040296 17941714 PMC2020495

[7] Hatzis C, Pusztai L, Valero V, Booser DJ, Esserman L, Lluch A, et al. A genomic predictor of response and survival following taxane-anthracycline chemotherapy for invasive breast cancer. JAMA. 2011;305(18):1873–81. doi: 10.1001/jama.2011.593 21558518 PMC5638042

[8] Miyake T, Nakayama T, Naoi Y, Yamamoto N, Otani Y, Kim SJ, et al. GSTP1 expression predicts poor pathological complete response to neoadjuvant chemotherapy in ER-negative breast cancer. Cancer Sci. 2012;103(5):913–20. doi: 10.1111/j.1349-7006.2012.02231.x 22320227 PMC7659189

[9] Chen JW, Russell RP, Desai T, Fiel-Gan M, Bhat V, Dias Gaui MF. RNA expression classifiers from a model of breast epithelial cell organization to predict pathological complete response in triple negative breast cancer. Arch Clin Biomed Res. 2021;5(3):376–95. doi: 10.26502/acbr.50170170

[10] Gianni L, Huang CS, Egle D, Bermejo B, Zamagni C, Thill M, et al. Pathologic complete response (pCR) to neoadjuvant treatment with or without atezolizumab in triple-negative, early high-risk and locally advanced breast cancer: NeoTRIP Michelangelo randomized study. Ann Oncol. 2022;33(5):534–43. doi: 10.1016/j.annonc.2022.02.004 35182721

[11] Dugo M, Huang C-S, Egle D, Bermejo B, Seitz RS, Nielsen TJ, et al. Longitudinal transcriptomic profiling identifies predictors of response to neoadjuvant chemoimmunotherapy in triple-negative breast cancer: results from the NeoTRIPaPDL1 trial. Ann Oncol. 2026;37(9):1278–92. doi: 10.1016/j.annonc.2026.05.694 42162664

[12] Wu SZ, Al-Eryani G, Roden DL, Junankar S, Harvey K, Andersson A, et al. A single-cell and spatially resolved atlas of human breast cancers. Nat Genet. 2021;53(9):1334–47. doi: 10.1038/s41588-021-00911-1 34493872 PMC9044823

[13] Wolf DM, Yau C, Wulfkuhle J, Brown-Swigart L, Gallagher RI, Lee PRE, et al. Redefining breast cancer subtypes to guide treatment prioritization and maximize response: Predictive biomarkers across 10 cancer therapies. Cancer Cell. 2022;40(6):609-623.e6. doi: 10.1016/j.ccell.2022.05.005 35623341 PMC9426306

[14] Liberzon A, Birger C, Thorvaldsdóttir H, Ghandi M, Mesirov JP, Tamayo P. The Molecular Signatures Database (MSigDB) hallmark gene set collection. Cell Syst. 2015;1(6):417–25. doi: 10.1016/j.cels.2015.12.004 26771021 PMC4707969

[15] Yoshihara K, Shahmoradgoli M, Martínez E, Vegesna R, Kim H, Torres-Garcia W, et al. Inferring tumour purity and stromal and immune cell admixture from expression data. Nat Commun. 2013;4:2612. doi: 10.1038/ncomms3612 24113773 PMC3826632

[16] Knapp G, Hartung J. Improved tests for a random effects meta-regression with a single covariate. Stat Med. 2003;22(17):2693–710. doi: 10.1002/sim.1482 12939780

[17] Röver C, Knapp G, Friede T. Hartung-Knapp-Sidik-Jonkman approach and its modification for random-effects meta-analysis with few studies. BMC Med Res Methodol. 2015;15:99. doi: 10.1186/s12874-015-0091-1 26573817 PMC4647507

[18] Newman AM, Steen CB, Liu CL, Gentles AJ, Chaudhuri AA, Scherer F, et al. Determining cell type abundance and expression from bulk tissues with digital cytometry. Nat Biotechnol. 2019;37(7):773–82. doi: 10.1038/s41587-019-0114-2 31061481 PMC6610714

[19] Becht E, Giraldo NA, Lacroix L, Buttard B, Elarouci N, Petitprez F, et al. Estimating the population abundance of tissue-infiltrating immune and stromal cell populations using gene expression. Genome Biol. 2016;17:218. doi: 10.1186/s13059-016-1070-5 PMC5073889 27765066

[20] Benjamini Y, Hochberg Y. Controlling the false discovery rate: A practical and powerful approach to multiple testing. Journal of the Royal Statistical Society Series B: Statistical Methodology. 1995;57(1):289–300. doi: 10.1111/j.2517-6161.1995.tb02031.x

