# Supplementary material for: Patient overlap, cohort-relative scaling, and biological context in cross-cohort evaluation of a mitochondrial proteostasis-associated score in triple-negative breast cancer
Source: PLoS One. 2026 Oct 9;21(10):e0355652. doi: 10.1371/journal.pone.0355652 (PMC13652229; doi:10.1371/journal.pone.0355652)
Supplement: Supplementary file 1 [file pone.0355652.s001.pdf]

# S1 Appendix. Supplementary methods, tables, and figures

## Patient overlap, cohort-relative scaling, and biological context in cross-cohort evaluation of a mitochondrial proteostasis-associated score in triple-negative breast cancer

Xiaoyuan Weng, MD | Quanzhou Medical College | Corresponding author: 2016034@qzmc.edu.cn

## Supplementary methods

### Analysis hierarchy and units

The primary clinical estimand was the association between the primary cohort-relative 15-gene score and source-study-defined pCR. Four accession-defined cohorts formed the primary meta-analytic structure. The three-principal-study-source analysis, Paule-Mandel tau-squared estimation, and single RankScore implementation were sensitivity analyses.

### Patient-overlap assessment

Public GSE20194 metadata were screened using source patient identifiers and strict TNBC plus binary pCR/RD eligibility. Seventy-one records mapped to 68 unique patients after collapsing three R1 repeats. Fifty-eight identifiers were present in GSE25055; 57 were strict-TNBC in both accessions, one had discordant PR annotation, and 10 were unmatched. Clinical concordance and expression similarity were secondary checks, not matching criteria.

### Score construction and reference scope

Target-gene expression was standardized against the full processed expression-matrix sample range for each accession before forming the equal-weight 15-gene composite; the composite was then restandardized within the final analysis cohort. This reference dependence is part of the primary score definition.

### Fixed RankScore sensitivity

The sensitivity retained the same 15 genes and positive direction but ranked expression within each sample against a fixed 12,151-gene universe. RankScore = [mean rank of the 15 target genes - (15+1)/2]/(G-15), with G=12,151; ranks are ascending and ties receive their average rank. The gene set, missing-gene rule, and background universe were applied uniformly across cohorts. Effects were reported per +0.10 RankScore and per within-cohort RankScore SD.

### Meta-analysis and study-source sensitivity

Cohort-specific log-odds ratios were pooled by REML random-effects meta-analysis with modified Hartung-Knapp inference. The zero boundary for tau-squared was explicitly evaluated. Paule-Mandel tau-squared with the same modified Hartung-Knapp inference was the estimator sensitivity. For the three-source analysis, GSE25055 and GSE25065 retained accession-specific intercepts but contributed one shared score slope.

### LOCO residual diagnostic

For each held-out cohort, nuisance slopes linking the primary score to proliferation, immune, and stromal scores were estimated in the other cohorts without using the held-out outcome. Held-out residuals were scaled by the training residual SD. The residual-only outcome model estimates a different association from the direct multivariable score model. A leave-one-study-source-out version held GSE25055 and GSE25065 out together.

### NeoTRIP analysis

The raw 15-gene NeoTRIP score used the provider-supplied linear-scale matrix, with all 401 profiles as the gene/composite standardization reference; all target-gene values were nonnegative in the processed matrix. The retained 150-patient paired values and E2F/G2M/ESTIMATE-derived calculations are reported as contextual analyses.

## Single-cell analysis

The 10-TNBC subset of GSE176078 contains 42,512 cells with source-provided patient and cell-type annotations; `Cancer Epithelial` is the source label used for malignant epithelium. Fig 5b required one malignant-epithelial row and non-cancer rows with positive pooled counts. Eight patients met this rule; CID3946 and CID44041 lacked a malignant-epithelial row. Non-cancer counts were pooled within patient before CP10K normalization, log1p transformation, and 15-gene averaging.

## I-SPY2 analysis

The public GSE194040 resource contains processed expression and relevant clinical variables. The 363-patient HR-negative/HER2-negative analysis included treatment-arm-adjusted, arm+MammaPrint, and score-by-arm interaction models. Because I-SPY2 differs from the strict-TNBC primary cohorts in receptor definition and treatment structure, these analyses are reported as contextual.

## Immune analyses

CIBERSORTx and MCP-counter analyses are retained as exploratory context. Full machine-readable correlation, joint-model, quality, and concordance tables are supplied in S2. No score-lineage correlation or cell-adjusted pCR association remained significant after the prespecified within-family false-discovery-rate correction.

### Exploratory-analysis inventory and multiplicity

Analyses were classified as follows: four-cohort raw-score REML+mKH primary analysis; three-source and Paule-Mandel source/estimator sensitivities; one RankScore implementation sensitivity; same-cohort proliferation/tissue-context and residual models as exploratory decomposition; LOCO and leave-one-source-out analyses as nuisance-relation diagnostics; immune deconvolution families with prespecified within-family Benjamini-Hochberg correction; and NeoTRIP, single-cell, and I-SPY2 as contextual analyses.

### Cohort-specific logistic inference and small-sample handling

Retained cohort-specific outcome models used ordinary binomial logistic generalized linear models with maximum-likelihood coefficients and Wald 95% confidence intervals; no Firth or other small-sample penalization was applied. The All retained cohort-by-model fits converged without complete separation. Estimates from the smallest cohort (GSE32646; n=26, 10 pCR events) are imprecise. The continuous score was the primary specification; the quadratic-term sensitivity is reported in Table S12.

### Correlation uncertainty and meta-analysis

For cohort-specific score-feature correlations, 95% confidence intervals were calculated by Fisher z transformation with standard error 1/sqrt(n-3), then back-transformed to the correlation scale. For pooled correlation summaries, cohort correlations were transformed with atanh(r), assigned variance 1/(n-3), pooled by REML with modified Hartung-Knapp inference, and back-transformed with tanh. These intervals are approximate for Spearman correlations and are interpreted descriptively.

## Supplementary tables

### Table S1. Cohort, platform, treatment, and source-defined pCR characteristics.

| Cohort | Parent study/source | Platform | Pretreatment specimen | Neoadjuvant treatment | pCR definition retained for this analysis | N | pCR | RD |
|---|---|---|---|---|---|---|---|---|
| GSE25055 | Hatzis et al. 2011; GSE25066 SuperSeries; discovery cohort | GPL96 — Affymetrix Human Genome U133A Array | Tumor biopsy before systemic therapy; parent discovery cohort used FNA (MDACC) or core biopsy (I-SPY-1). | Sequential taxane- and anthracycline-based chemotherapy; regimen varied within the multicenter parent study. Endocrine therapy, where applicable to ER-positive disease, is irrelevant to the strict-TNBC subset. | Source binary pCR vs residual disease retained. Parent article describes pCR as no invasive or metastatic breast cancer identified after neoadjuvant chemotherapy. | 112 | 37 | 75 |
| GSE25065 | Hatzis et al. 2011; GSE25066 SuperSeries; validation cohort | GPL96 — Affymetrix Human Genome U133A Array | Tumor biopsy before systemic therapy; parent validation cohort used FNA or core biopsy across participating sites. | Sequential taxane- and anthracycline-based chemotherapy; regimen varied within the multicenter parent study. Endocrine therapy, where applicable to ER-positive disease, is irrelevant to the strict-TNBC subset. | Source binary pCR vs residual disease retained. Parent article describes pCR as no invasive or metastatic breast cancer identified after neoadjuvant chemotherapy. | 58 | 20 | 38 |
| GSE32646 | Miyake et al. 2012, Osaka University | GPL570 — Affymetrix Human Genome U133 Plus 2.0 Array | Fresh-frozen tumor obtained by vacuum-assisted core biopsy before P-FEC. | Paclitaxel followed by FEC (5-fluorouracil/epirubicin/cyclophosphamide); source regimen was weekly paclitaxel followed by four 3-weekly FEC cycles. | No evidence of residual invasive cancer in both breast and axilla. | 26 | 10 | 16 |
| GSE163882 | Chen et al. 2021, multicenter retrospective cohort | GPL18573 — Illumina NextSeq 500 | FFPE pretreatment breast tumor biopsy; lymph-node biopsy when available in the parent study. | Neoadjuvant anthracycline–taxane-based chemotherapy; parent report describes standard NAC incorporating a taxane, anthracycline, and cyclophosphamide, with regimen determined by treating physicians/institutions. | pCR and residual disease were defined by the tissue block’s institution of origin in the pathology report; the source annotation was retained. | 89 | 37 | 52 |

### Table S2. Model-level patient and pCR-event accounting.

| Analysis | GSE25055 n/pCR | GSE25065 n/pCR | GSE32646 n/pCR | GSE163882 n/pCR | Total n/pCR | Note |
|---|---|---|---|---|---|---|
| Raw score (primary) | 112/37 | 58/20 | 26/10 | 89/37 | 285/104 | No attrition |
| Score + proliferation (exploratory) | 112/37 | 58/20 | 26/10 | 89/37 | 285/104 | No covariate-related attrition |
| Score + proliferation + immune + stromal (exploratory) | 112/37 | 58/20 | 26/10 | 89/37 | 285/104 | No covariate-related attrition |
| Within-cohort residual (post hoc) | 112/37 | 58/20 | 26/10 | 89/37 | 285/104 | No attrition |
| Proliferation only (contextual) | 112/37 | 58/20 | 26/10 | 89/37 | 285/104 | No attrition |
| LOCO residual | 112/37 | 58/20 | 26/10 | 89/37 | 285/104 | Each row is the held-out fold; pooled across all 4 folds |
| Random-slope LOCO sensitivity | 112/37 | 58/20 | 26/10 | 89/37 | 285/104 | One-stage model on all 285 patients |
| Fixed single-sample rank-score sensitivity (0.10-unit) | 112/37 | 58/20 | 26/10 | 89/37 | 285/104 | Same retained patient set |

### Table S3. Patient-overlap counterfactual using 57 strict-to-strict duplicates.

| Scenario | k | Nominal n | Nominal pCR | OR (95% CI) | P | tau² | I² (%) | Interpretation |
|---|---|---|---|---|---|---|---|---|
| Primary analysis; GSE20194 excluded | 4 | 285 | 104 | 0.9583 (0.6392–1.437) | 0.7602 | | 0 | Primary 285-patient analysis; no duplicated GSE20194 records added. |
| Naive duplicate-as-fifth-cohort counterfactual | 5 | 342 | 126 | 0.9705 (0.7047–1.337) | 0.8081 | 5.554e-06 | 0 | Counterfactual only; double-counts 57 patients already represented in GSE25055 and creates false nominal precision. |
| GSE20194 strict-overlap duplicate subset only | 1 | 57 | 22 | 1.028 (0.6034–1.752) | 0.9187 | | | Descriptive effect among the 57 strict-to-strict identifier-matched duplicate patients. |

### Table S4. Four-cohort and three-source meta-analysis with REML and Paule-Mandel tau-squared estimators.

| Analysis | tau² estimator | k | df | n | pCR | OR (95% CI) | P | tau² | I² (%) |
|---|---|---|---|---|---|---|---|---|---|
| Four cohorts | REML | 4 | 3 | 285 | 104 | 0.9583 (0.6392–1.437) | 0.7602 | 0 | 0 |
| Four cohorts | Paule-Mandel | 4 | 3 | 285 | 104 | 0.9583 (0.6392–1.437) | 0.7602 | 0 | 0 |
| Three sources | REML | 3 | 2 | 285 | 104 | 0.9708 (0.4924–1.914) | 0.8683 | 0.02012 | 13.68 |
| Three sources | Paule-Mandel | 3 | 2 | 285 | 104 | 0.9645 (0.5296–1.757) | 0.8198 | 0.007172 | 13.68 |

### Table S5. Fixed single-sample RankScore sensitivity.

| Analysis | Unit | Method | k | n | pCR | OR (95% CI) | P | tau² | I² (%) |
|---|---|---|---|---|---|---|---|---|---|
| Four cohorts | Per 0.10 RankScore | REML+mKH | 4 | 285 | 104 | 0.6134 (0.148–2.542) | 0.3539 | 0 | 0 |
| Four cohorts | Per 0.10 RankScore | PM+mKH | 4 | 285 | 104 | 0.6134 (0.148–2.542) | 0.3539 | 0 | 0 |
| Three sources | Per 0.10 RankScore | REML+mKH | 3 | 285 | 104 | 0.6123 (0.0899–4.17) | 0.386 | 0 | 0 |
| Three sources | Per 0.10 RankScore | PM+mKH | 3 | 285 | 104 | 0.6123 (0.0899–4.17) | 0.386 | 0 | 0 |
| Four cohorts | Per 1 SD RankScore | REML+mKH | 4 | 285 | 104 | 0.8682 (0.5853–1.288) | 0.3368 | 0 | 0 |
| Four cohorts | Per 1 SD RankScore | PM+mKH | 4 | 285 | 104 | 0.8682 (0.5853–1.288) | 0.3368 | 0 | 0 |
| Three sources | Per 1 SD RankScore | REML+mKH | 3 | 285 | 104 | 0.8678 (0.5099–1.477) | 0.37 | 0 | 0 |
| Three sources | Per 1 SD RankScore | PM+mKH | 3 | 285 | 104 | 0.8678 (0.5099–1.477) | 0.37 | 0 | 0 |

### Table S6A. Four-fold LOCO held-out outcome associations.

| Held-out cohort | n | pCR | OR | 95% CI | P |
|---|---|---|---|---|---|
| GSE163882 | 89 | 37 | 1.19 | 0.8304–1.706 | 0.3431 |
| GSE25055 | 112 | 37 | 0.681 | 0.4657–0.9958 | 0.04753 |
| GSE25065 | 58 | 20 | 0.6345 | 0.3756–1.072 | 0.08899 |
| GSE32646 | 26 | 10 | 0.5765 | 0.2245–1.48 | 0.2523 |

**Table S6B. LOCO held-out scale and background-correlation diagnostics.**

| heldout_cohort | train_n | training_R2 | train_residual_sd | test_residual_sd_in_training_SD_units | pearson_proliferation_z | pearson_immune_z | pearson_stromal_z |
|---|---|---|---|---|---|---|---|
| GSE163882 | 196 | 0.3506 | 0.8059 | 1.226 | 0.5471 | 0.4698 | 0.6247 |
| GSE25055 | 173 | 0.3957 | 0.7774 | 1.133 | -0.1477 | -0.1573 | -0.2683 |
| GSE25065 | 227 | 0.3743 | 0.791 | 1.122 | -0.2101 | -0.07765 | -0.09053 |
| GSE32646 | 259 | 0.3338 | 0.8162 | 0.9194 | 0.1239 | 0.09871 | -0.1442 |

**Table S7. Leave-one-study-source-out residual diagnostic.**

| Source | Held-out accession(s) | n/pCR | OR (95% CI) | P | Train residual SD | Held-out SD / train SD | Pearson r: prolif; immune; stromal |
|---|---|---|---|---|---|---|---|
| MDACC_GSE25055_GSE25065 | GSE25055+GSE25065 | 170/57 | 0.719 (0.554-0.934) | 0.013 | 0.696 | 1.321 | -0.203; -0.117; -0.248 |
| GSE32646 | GSE32646 | 26/10 | 0.576 (0.225-1.480) | 0.252 | 0.816 | 0.919 | 0.124; 0.099; -0.144 |
| GSE163882 | GSE163882 | 89/37 | 1.190 (0.830-1.706) | 0.343 | 0.806 | 1.226 | 0.547; 0.470; 0.625 |

Table S8. Score reference-scope diagnostic.

| cohort | n_retained_patients | gene_z_reference | final_composite_reference | pearson_r_vs_reference | RMSE_vs_reference | max_abs_delta_vs_reference | interpretation |
|---|---|---|---|---|---|---|---|
| GSE32646 | 26 | all 115 samples in public GSE32646 Series Matrix | 26 retained TNBC patients | 1 | 1.944e-10 | 4.319e-10 | Public-input reconstruction confirms accession-wide gene standardization followed by analysis-cohort composite re-standardization. |

Table S9. NeoTRIP score-reference comparison.

| label | transform | gene_reference | composite_reference | n | mean_delta | ci_low | ci_high | t_p | pearson_r_vs_reference | RMSE_vs_reference |
|---|---|---|---|---|---|---|---|---|---|---|
| reported_pair_values | reported | not specified | not specified | 150 | -0.8941 | -1.05 | -0.7384 | 6.699e-22 | 1 | 0 |
| raw_all401 | raw | all401 | all401 | 150 | -0.8941 | -1.05 | -0.7384 | 6.699e-22 | 1 | 1.546e-16 |
| cliplog_allbase241 | cliplog | allbase241 | allbase241 | 150 | -1.028 | -1.198 | -0.8584 | 1.628e-23 | 0.9276 | 0.4149 |
| raw_allbase241 | raw | allbase241 | allbase241 | 150 | -0.8694 | -1.021 | -0.7173 | 9.177e-22 | 0.9999 | 0.03581 |

Table S10. Paired malignant-epithelial versus pooled-count non-cancer comparisons.

| Patient | Cancer Epithelial row | Cancer n | Non-cancer n | Paired eligible | Reason if excluded | Cancer score | Pooled non-cancer score | Difference |
|---|---|---|---|---|---|---|---|---|
| CID3946 | no | | 774 | no | No Cancer Epithelial row in processed table | | | |
| CID3963 | yes | 222 | 3305 | yes | | 0.7059 | 0.6187 | 0.0873 |
| CID44041 | no | | 2131 | no | No Cancer | | | |

| Patient | Cancer Epithelial row | Cancer n | Non-cancer n | Paired eligible | Reason if excluded | Cancer score | Pooled non-cancer score | Difference |
|---|---|---|---|---|---|---|---|---|
| | | | | | Epithelial row in processed table | | | |
| CID4465 | yes | 124 | 1440 | yes | | 0.8112 | 0.5374 | 0.2738 |
| CID4495 | yes | 1184 | 6801 | yes | | 0.8195 | 0.4295 | 0.3900 |
| CID44971 | yes | 894 | 7092 | yes | | 0.8817 | 0.5478 | 0.3339 |
| CID44991 | yes | 4018 | 3005 | yes | | 0.7197 | 0.3663 | 0.3534 |
| CID4513 | yes | 1058 | 4561 | yes | | 0.8091 | 0.5625 | 0.2466 |
| CID4515 | yes | 2169 | 1980 | yes | | 0.7453 | 0.5385 | 0.2068 |
| CID4523 | yes | 1167 | 587 | yes | | 0.6699 | 0.5194 | 0.1505 |

Table S11. I-SPY2 contextual analysis summary.

| Component | Object | Supporting material | Analysis status | Role in study |
|---|---|---|---|---|
| Public processed expression input | GSE194040 normalized/batch-corrected gene-level matrix | Public GEO files and score-construction resources documented in S2. | Public processed input documented in S2. | Contextual source; outside the primary strict-TNBC cohorts. |
| Patient-level I-SPY2 derived table | HR, HER2, pCR, MammaPrint, arm, fixed-score value for each analyzed patient | S2 patient-level I-SPY2 table and workflow. | 363-patient analysis table available in S2. | Contextual analysis; outside the primary meta-analysis. |
| I-SPY2 score construction | 15-gene score for HR-/HER2- subset | S2 score-construction code and named public GSE194040 inputs. | Score construction documented and reproducible. | Contextual analysis using the same 15-gene score framework. |
| Unadjusted I-SPY2 effect | n=363, events=142, OR=0.784121, P=0.028604 | Patient-level table and I-SPY2 model output. | Patient-level model reproducible from S2 inputs. | Contextual unadjusted association. |
| Treatment-arm-adjusted I-SPY2 effect | n=363, events=142, OR=0.715711, 95% CI 0.566521-0.904189, P=0.005040 | S2 patient-level model and output. | Patient-level model reproducible from S2 inputs. | Contextual adjusted association. |
| Arm + MammaPrint sensitivity | OR=0.694933, 95% CI 0.547590-0.881922, P=0.002758 | S2 patient-level model and output. | Patient-level model reproducible from S2 inputs. | Contextual sensitivity analysis. |
| Score-by-arm interaction | omnibus LRT P=0.233146 | S2 patient-level interaction model and output. | Patient-level model reproducible from S2 inputs. | Contextual treatment-interaction analysis. |
| Expanded 4-cohort + unadjusted I-SPY2 pool | OR=0.867184, P=0.208435 | Aggregate effect rows and I-SPY2 patient-level estimate. | Aggregate estimate reproduced from the stated components. | Contextual comparison outside the primary four-cohort meta-analysis. |

Table S12. Score-linearity sensitivity.

| analysis | cohort | n | events | quadratic_beta | quadratic_SE | quadratic_Wald_P | quadratic_LRT_P |
|---|---|---|---|---|---|---|---|
| Pooled with fixed cohort intercepts | All four cohorts | 285 | 104 | -0.09286 | 0.08776 | 0.29 | 0.2644 |
| Cohort-specific exploratory | GSE163882 | 89 | 37 | -0.476 | 0.2938 | 0.1052 | 0.04233 |
| Cohort-specific exploratory | GSE25055 | 112 | 37 | 0.1016 | 0.1426 | 0.476 | 0.4824 |
| Cohort-specific exploratory | GSE25065 | 58 | 20 | 0.519 | 0.2815 | 0.06516 | 0.06028 |
| Cohort-specific exploratory | GSE32646 | 26 | 10 | -1.201 | 0.6846 | 0.07948 | 0.02505 |

## Supplementary figures

Fig S1. Raw-score distributions by response

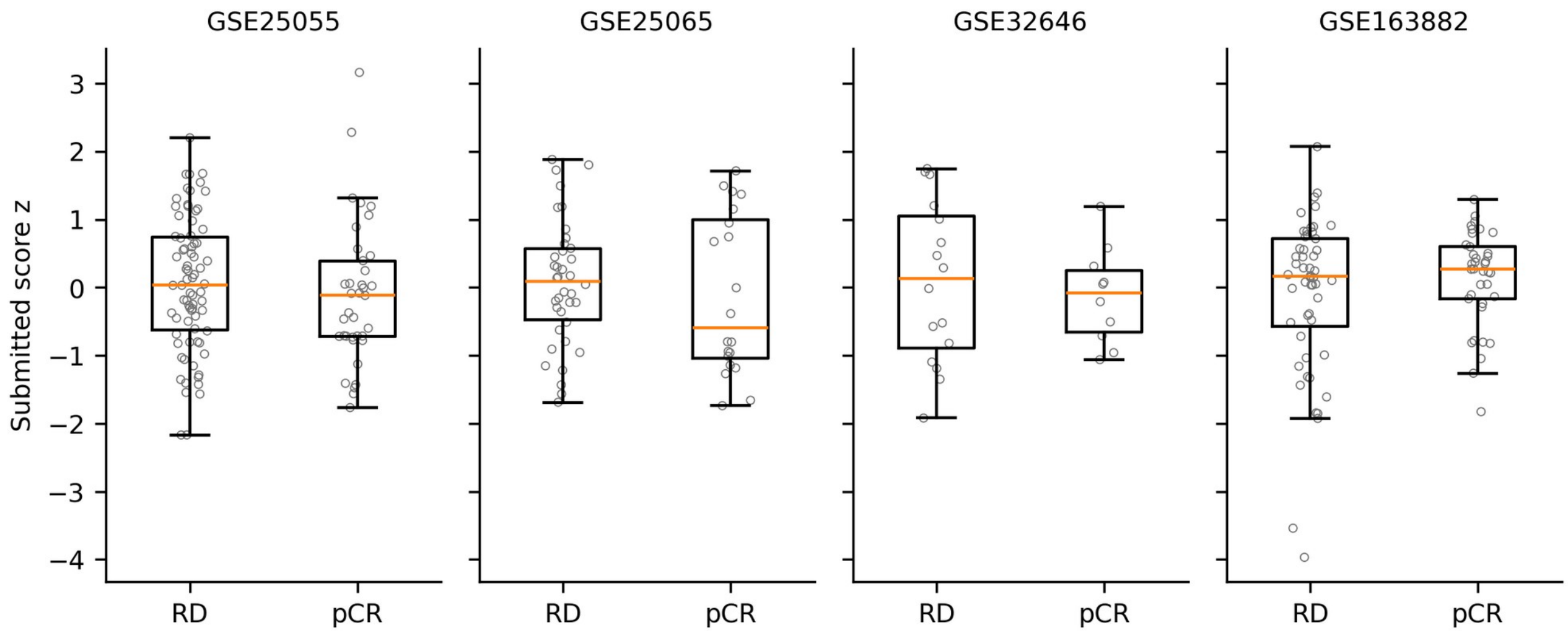


Fig S1. Cohort-specific primary raw-score distributions by pCR status. Patients are the inferential units; boxes show median and interquartile range.

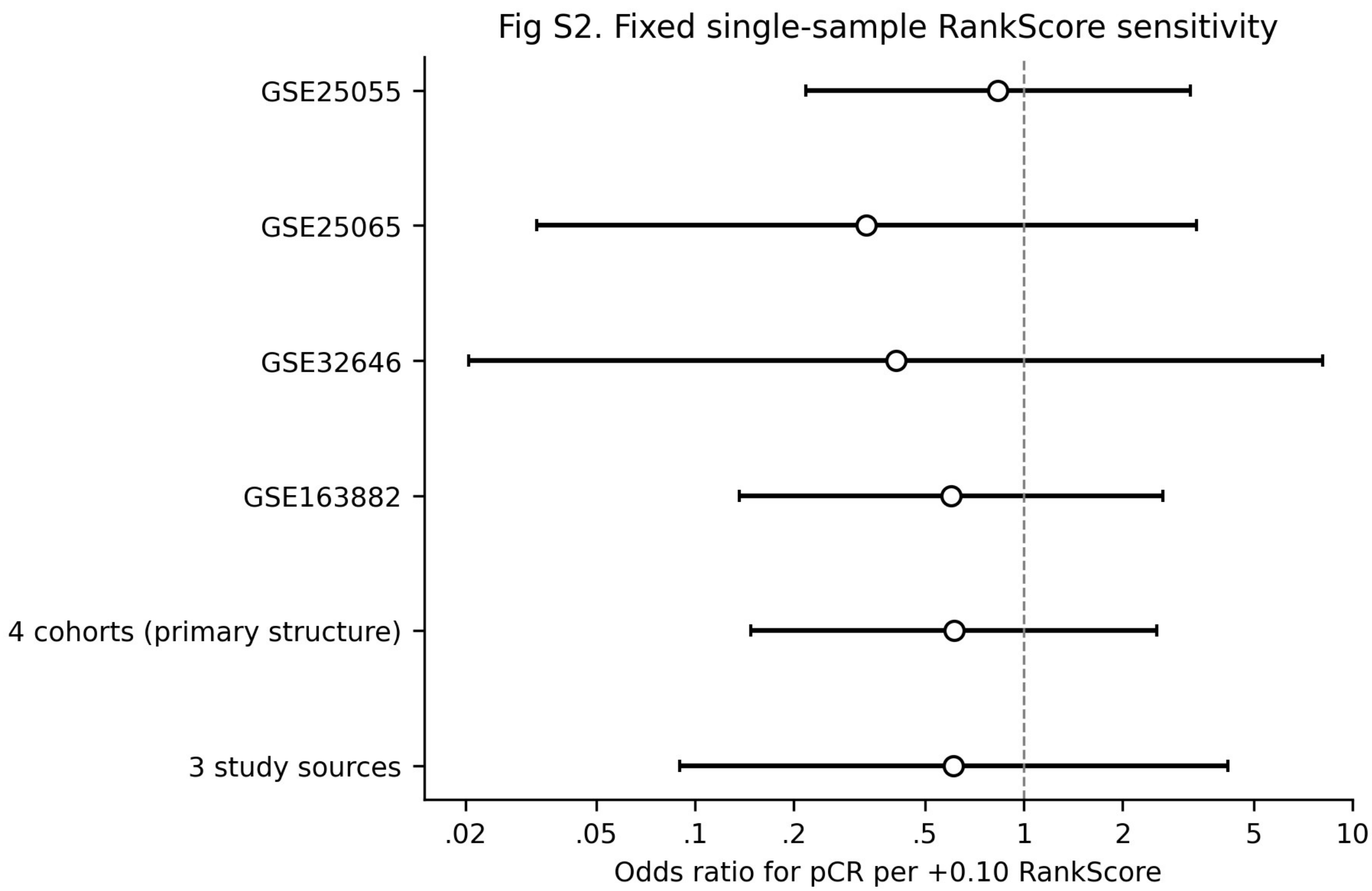


Fig S2. Fixed single-sample RankScore sensitivity. Points and 95% confidence intervals are shown per +0.10 RankScore. The three-source pooled row is a sensitivity analysis.

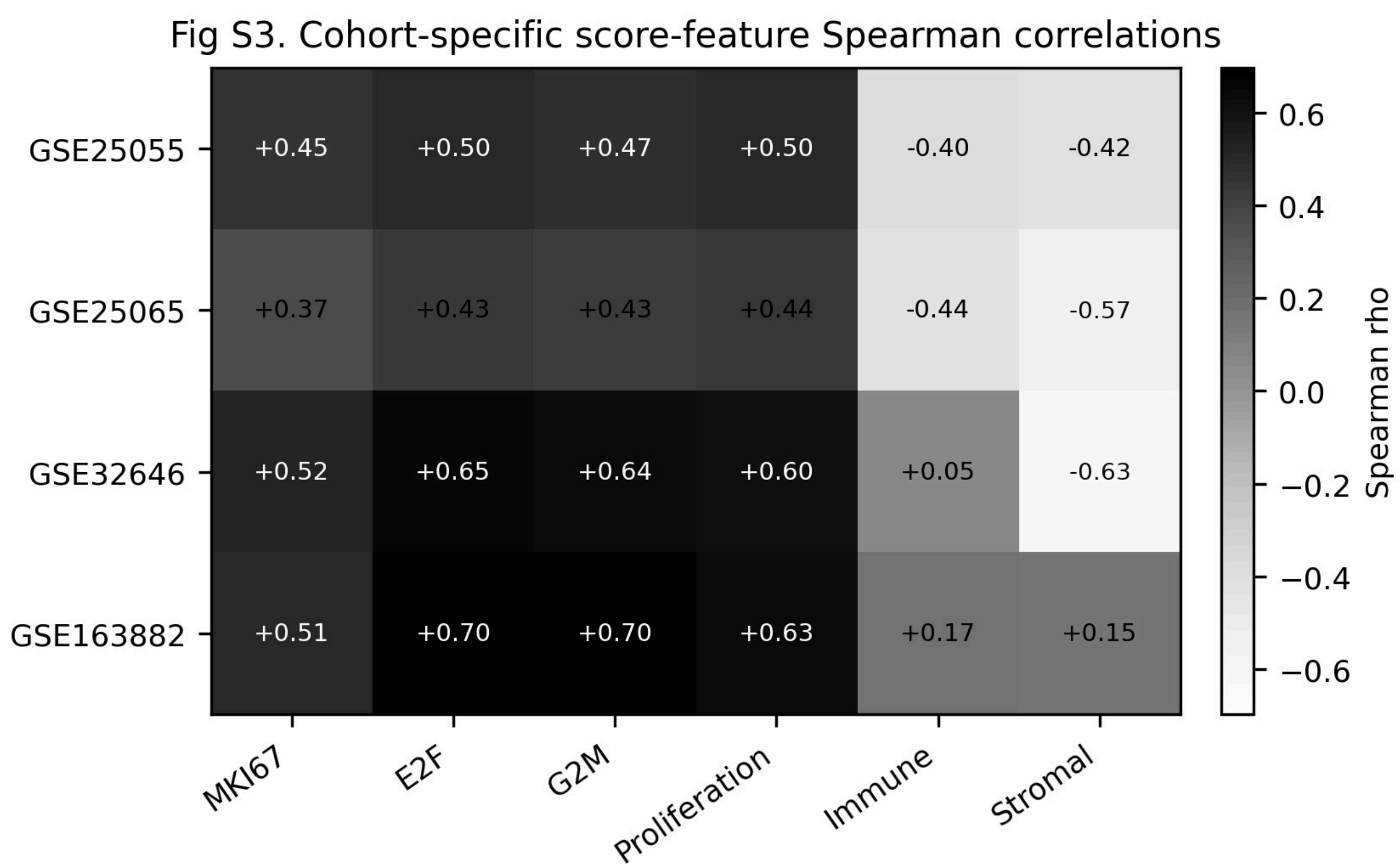


Fig S3. Cohort-specific Spearman correlations of the primary score with proliferation and tissue-context features. Correlations are descriptive/exploratory and vary across cohorts.

## Fig S4. LOCO held-out diagnostics

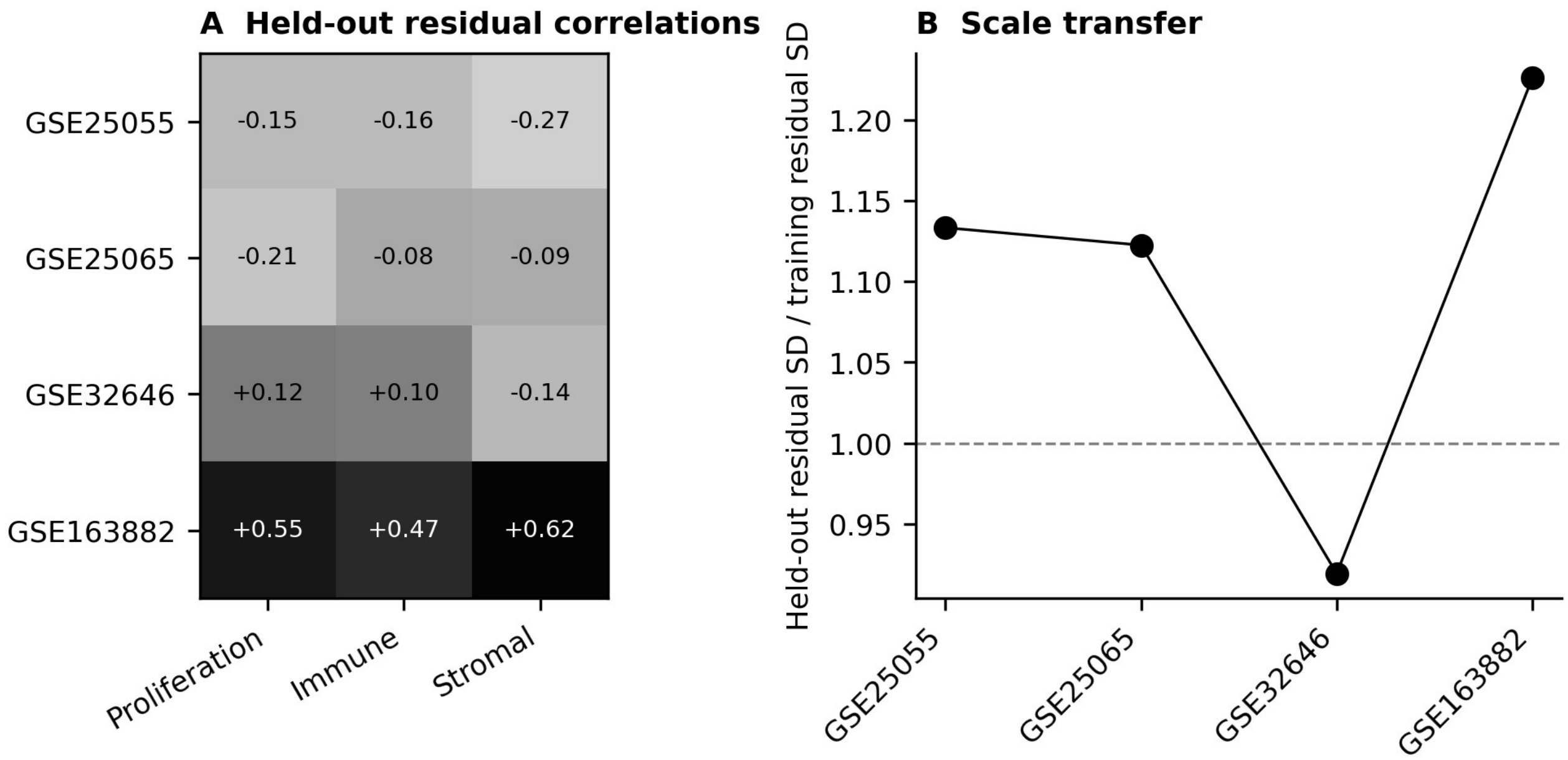


Fig S4. LOCO held-out diagnostics. Panel A shows Pearson correlations between the externally trained residual and held-out background variables. Panel B shows held-out residual SD expressed in training-residual-SD units; values differing from 1 indicate scale transfer differences.

## Fig S5. Immune deconvolution quality and concordance

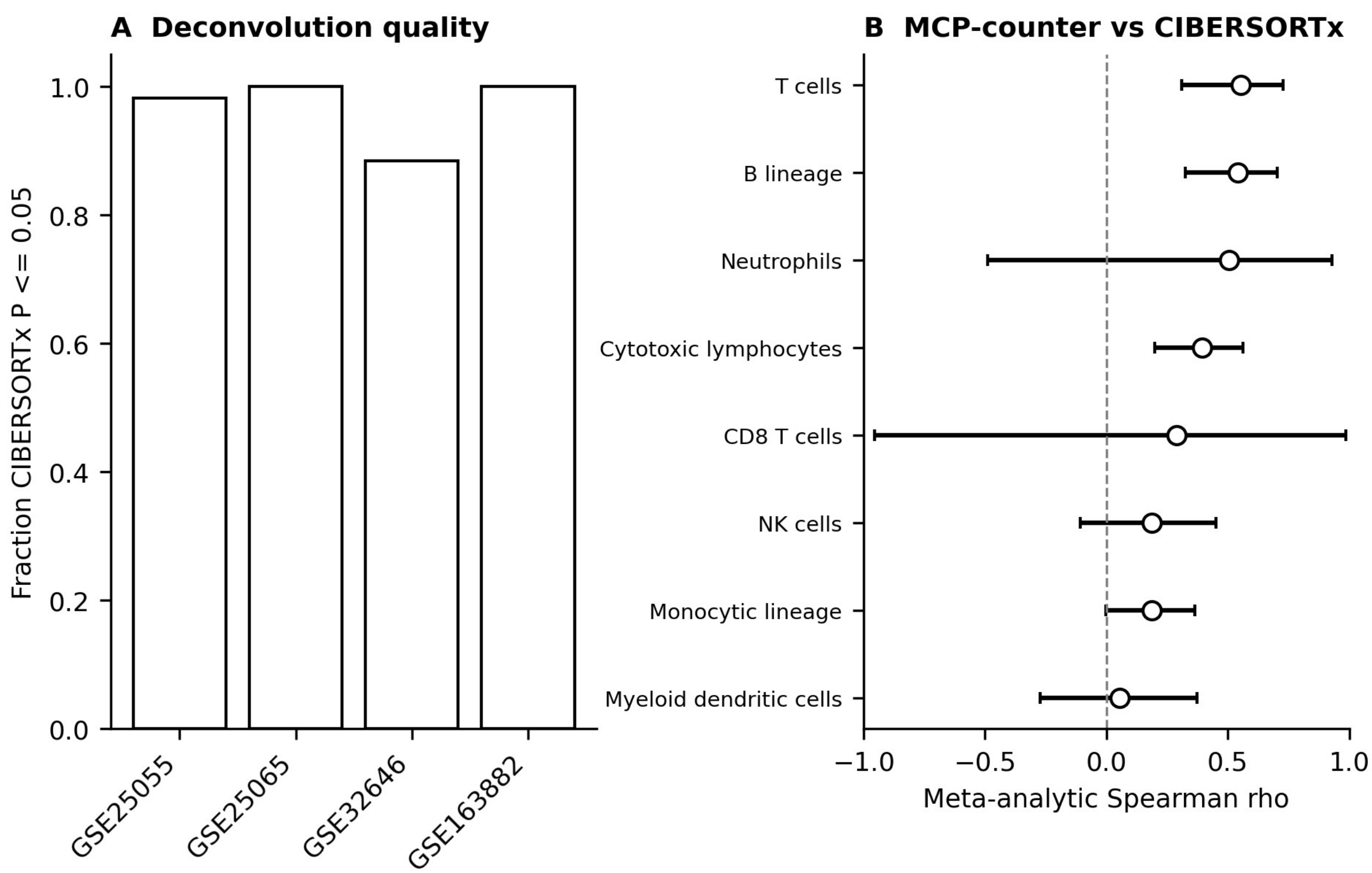


Fig S5. Immune deconvolution quality and broad lineage concordance. These analyses are exploratory.
