# Supplementary material for: Patient overlap, cohort-relative scaling, and biological context in cross-cohort evaluation of a mitochondrial proteostasis-associated score in triple-negative breast cancer
Source: PLoS One. 2026 Oct 9;21(10):e0355652. doi: 10.1371/journal.pone.0355652 (PMC13652229; doi:10.1371/journal.pone.0355652)
Supplement: Supplementary file 2 [file pone.0355652.s003.pdf]

<u>Reviewer Comments</u>

Overall recommendations: Major Revision

This manuscript addresses an important issue in transcriptomic biomarker validation, particularly patient overlap between public datasets and the influence of cohort-specific normalization and biological factors. The study is interesting; however, several methodological and interpretational issues should be clarified before the manuscript can be considered for publication.

Comment 1: The manuscript states that GSE25055 and GSE25065 originated from the same parent study. Please provide more justification for treating them as separate cohorts in the primary meta-analysis. It would also be helpful to explain whether this shared origin could affect the independence of the results.

Comment 2: The score was calculated using within-cohort standardization. Please explain more clearly why this normalization method was selected and discuss its limitations.

Comment 3: The score showed a strong correlation with proliferation. However, the raw score was not associated with pCR, while some adjusted analyses showed a significant association. Please explain this result more clearly. It would also be helpful to provide information about the correlation between the score and the other variables used in the adjustment models.

Comment 4: The leave-one-cohort-out (LOCO) residualization procedure is central to the study. However, the methodology is difficult to follow. Please provide a clearer step-by-step explanation of the procedure, preferably with a schematic figure or workflow.

Comment 5: The authors set negative ComBat-adjusted values to zero before log transformation. Please provide a stronger justification for this approach.

Comment 6: The included datasets may differ in patient characteristics, treatment regimens, sample processing, and definitions of pCR. Please provide a clear table comparing these characteristics across the primary cohorts. This will help readers understand the possible sources of differences between cohorts.

Comment 7: Please clearly report the definition of pathological complete response used in each dataset. Were the same criteria used in all cohorts? If the definitions were different, please discuss whether this could affect the meta-analysis.

Comment 8: Please provide more detail about how the final 15 genes were selected from the mitochondrial proteostasis/chaperone pathway. Were all genes from the original pathway included? If not, please clearly explain the selection criteria and the reason for excluding other genes.

Comment 9: The single-cell analysis is interesting, but it includes a limited number of tumors. Please provide more details about how malignant and non-malignant cells were identified and how the pseudobulk analysis was performed. The limitations of the small sample size should also be emphasized.

Comment 10: The authors provide scripts and derived data, which is appreciated. However, the manuscript states that not all upstream preprocessing steps can be fully reproduced. Please consider providing a more complete workflow from the original public datasets to the final results

Comment 11: The Introduction provides a useful overview of the challenges of cross-cohort transcriptomic biomarker validation. However, the specific knowledge gap addressed by this study should be stated more clearly.

Comment 12: The biological rationale for selecting a mitochondrial proteostasis-associated gene score in relation to TNBC treatment response and pCR requires further explanation.

Comment 13: The manuscript should clarify whether the primary objective is to evaluate a specific mitochondrial gene score or to demonstrate methodological challenges in public transcriptomic biomarker validation.

Comment 14: The identification of overlapping patients is an important aspect of this study. However, please provide more detail about how duplicate patients were identified and verified. Please clarify which variables or identifiers were used and how the authors confirmed that the identified samples represented the same patients rather than merely similar clinical characteristics.

Comment 15: Please provide additional justification for the selected random-effects meta-analysis approach. Because the primary analysis includes a limited number of cohorts, heterogeneity estimates may be unstable. It would be helpful to provide sensitivity analyses using alternative meta-analysis approaches or estimators.

Comment 16: Please provide a stronger justification for the use of within-cohort z-score normalization. Since this approach depends on the distribution of the cohort, the resulting score may not be directly applicable to an individual patient or an independent dataset without

recalculation. Please discuss this limitation clearly and, if possible, provide sensitivity analyses using an alternative scoring method.

Comment 17: The study combines datasets generated using different gene-expression platforms. Please provide a clearer description of the preprocessing steps applied to each dataset, including normalization, probe-to-gene mapping, handling of multiple probes representing the same gene, and treatment of missing genes. A summary table describing preprocessing for each dataset would improve reproducibility.

Comment 18: The final paragraph of introduction contains substantial methodological detail. Consider simplifying it and focusing more clearly on the research question and main objectives.
