# Supplementary material for: Patient overlap, cohort-relative scaling, and biological context in cross-cohort evaluation of a mitochondrial proteostasis-associated score in triple-negative breast cancer
Source: PLoS One. 2026 Oct 9;21(10):e0355652. doi: 10.1371/journal.pone.0355652 (PMC13652229; doi:10.1371/journal.pone.0355652)
Supplement: Supplementary file 3 [file pone.0355652.s004.docx]

**Response to Reviewers**

**Manuscript PONE-D-26-36973**

*Patient overlap, cohort-relative scaling, and biological context in cross-cohort evaluation of a mitochondrial proteostasis-associated score in triple-negative breast cancer*

We thank the Academic Editor and reviewers for their detailed evaluation. The revision corrects the patient-overlap analysis, reports the dependence structure at both four-accession and three-study-source levels, adds RankScore and Paule-Mandel sensitivities, clarifies the distinct roles of same-cohort adjustment and LOCO residualization, and strengthens the reproducibility materials. Re-examination of the archived analysis files also identified two Methods descriptions that did not match the executed analyses: the reference scope used to construct the primary cohort-relative score and the scale/reference used for the NeoTRIP raw score. The manuscript has been corrected accordingly; these corrections did not alter the primary four-cohort estimate. Page and line references below refer to the final clean revised manuscript with continuous line numbering.

# Summary of major revision outcomes

| Area | Revision |
| --- | --- |
| Primary inference retained | Four accession-defined cohorts remain the primary analysis: REML+mKH OR 0.958 (95% CI 0.639–1.437), P=0.760, tau²=0. |
| Study-source dependence | GSE25055 and GSE25065 are also summarized as one parent-study source; the three-source REML+mKH sensitivity is OR 0.971 (95% CI 0.492–1.914), P=0.868, tau²=0.0201. |
| Corrected overlap | The GSE20194 audit is corrected to 71 records → 68 unique patients; 58 identifier matches; 57 strict-TNBC in both accessions; one receptor-discordant match; 10 unmatched. The descriptive duplicate counterfactual is 285→342 nominal observations. |
| Score implementation | The submitted reference-scope description was corrected: target genes were standardized against the full processed matrix for each accession, followed by composite re-standardization in the analysis cohort. A fixed 12,151-gene single-sample RankScore sensitivity was added. |
| Reproducibility boundaries | Core clinical estimates can be re-fitted from patient-level derived data. S2 documents the corrected NeoTRIP workflow and provides executable contextual-analysis workflows and machine-readable outputs. |
| Submission files | A clean manuscript, a marked-up manuscript comparing the original submission with the final clean revision, revised S1 Appendix, S2 reproducibility archive, 600-dpi TIFF Figures 1–5, figure-source tables, and this point-by-point response are provided. |

# Methods-description corrections identified during revision

During revision, re-examination of the archived analysis files identified two Methods descriptions that did not match the executed analyses. For the primary score, target genes had been standardized across the full processed expression matrix for each accession, followed by re-standardization of the composite within the analysis cohort. For NeoTRIP, the reported raw score had been calculated directly from the provider-supplied linear-scale matrix using all 401 profiles as the standardization reference. We corrected the manuscript to reflect these executed analyses. The corrections did not alter the primary four-cohort estimate. The primary-score public-matrix reconstruction code and Methods specify the executed GSE163882 log2(TPM + 1) and duplicate-symbol maximum rules; these documentation corrections do not alter the archived patient scores or primary effect estimates. S2 distinguishes runnable patient-level workflows from historical NeoTRIP generator code requiring original project inputs.

# Academic Editor and journal requirements

**Journal requirement 1**

*Please ensure that your manuscript meets PLOS ONE style requirements, including file naming.*

Response: We reformatted and renamed the revision package consistently and provide separate clean and tracked manuscripts, S1 Appendix, S2 File, individual high-resolution figures, and the response letter. The manuscript title was also narrowed from “validation/transportability” language to “cross-cohort evaluation” to match the actual estimand.

Location in revised materials: Clean manuscript p. 1, lines 1-14; submission package filenames.

**Journal requirement 2**

*The Data Availability Statement should provide the repository name and DOI/accession number or direct link for each dataset.*

Response: The Data and Code Availability section now names the NCBI Gene Expression Omnibus and lists every accession used in the reported analyses: GSE25055, GSE25065, GSE32646, GSE163882, GSE194040, GSE319641, and GSE176078, with GSE20194 identified as an excluded overlapping accession. S2 additionally names the decisive public processed matrix files and platform annotation files.

Location in revised materials: Data and Code Availability, pp. 11-12, lines 246-252; S2 INPUT_MANIFEST.tsv and PREPROCESSING_PROTOCOL.md.

**Journal requirement 3**

*Please upload new copies of Figures 1 to 5 because the detail is not clear.*

Response: All five main figures were regenerated from source tables. The journal-supplied files are 600-dpi RGB TIFFs without alpha channels, use LZW compression, remain within the 7.5-inch maximum width and 10-MB file-size limit, and use figure text at 8 pt or larger. Figure 1 was simplified to separate the primary cohort path from the GSE20194 overlap audit; Figures 2-5 were similarly regenerated with larger labels and the revised evidence hierarchy.

Location in revised materials: Fig 1 caption, p. 12, lines 261-267; Fig 2 caption, p. 13, lines 283-287; Fig 3 caption, p. 15, lines 320-324; Fig 4 caption, p. 16, lines 339-341; Fig 5 caption, p. 16, lines 356-362; package Figures/Fig1.tif through Fig5.tif; figure source tables are included in S2 File under results/figure_source/.

**Journal requirement 4**

*Evaluate any reviewer-recommended citations for relevance; citation is not mandatory unless directed by the editor.*

Response: No reviewer required citation of a specific paper. In response to Reviewer 2's request for a clearer biological rationale, we added one directly relevant TNBC study showing adaptive mitochondrial oxidative-phosphorylation/fusion changes in chemotherapy-surviving TNBC models, while explicitly avoiding a causal claim for the selected chaperone genes. Other references remain focused on the clinical endpoint, public cohorts, MitoCarta pathway definition, reporting standards, meta-analytic methods, tissue-composition methods, NeoTRIP, and single-cell source data.

Location in revised materials: Biological rationale, p. 3, lines 53-59; reference 4, p. 20.

**Additional Editor requirement: point-by-point response**

*Provide a detailed point-by-point response addressing every comment and specify exact page and line numbers.*

Response: This document responds to each numbered and unnumbered substantive comment below. Page/line references correspond to the clean revised manuscript with continuous line numbering.

Location in revised materials: clean revised manuscript with final continuous line numbering; supporting files as cited in this response.

**Additional Editor requirement: clean and marked-up files**

*Upload both a clean revised manuscript and a marked-up copy highlighting all modifications.*

Response: We provide a clean revised manuscript and a marked-up Word comparison generated from the original submitted manuscript against the final clean revision. The marked file shows the original submitted text as deletions and the revised text as insertions; accepting all revisions yields the final clean manuscript, and rejecting all revisions restores the original submitted substantive manuscript.

Location in revised materials: clean revised manuscript with final continuous line numbering; supporting files as cited in this response.

**Additional Editor requirement: data and code integrity**

*Ensure underlying data, cohort definitions, and scaling code are clearly documented and accessible.*

Response: S2 provides patient-level derived data, named public processed inputs, preprocessing documentation, and executable workflows for the core cohort models, RankScore, source-level LOCO analysis, patient-level I-SPY2 and reduced-input single-cell analyses, together with machine-readable NeoTRIP outputs and historical NeoTRIP generator code that requires the original project inputs.

Location in revised materials: Data and Code Availability, pp. 11-12, lines 246-252; S2 README.md, INPUT_MANIFEST.tsv, PREPROCESSING_PROTOCOL.md, ANALYSIS_CHANGELOG.md, ANALYTICAL_HISTORY.md, code/, results/, and the contextual-analysis documentation described in the S2 README.

# Reviewer #1

**Comment 1**

*In the abstract, the authors need to mention the improvement of the proposed model as compared to the existing models.*

Response: The study evaluates a fixed candidate score rather than developing a new predictive model. The Abstract now states the fixed-score estimand, the four-cohort and three-source results, the fixed RankScore sensitivity, and the study-source precision directly. Model-improvement metrics would address a different model-development question.

Location in revised materials: Abstract, p. 2, lines 15-39.

**Comment 2**

*At the introduction, the key contributions of the proposed model should be concise and clearly provided.*

Response: We revised the Introduction to state the knowledge gap and contribution concisely: public accession labels do not guarantee participant independence, cohort-specific normalization creates reference dependence, and the paper evaluates one fixed mitochondrial score as a test case rather than proposing a new model.

Location in revised materials: Introduction, p. 3, lines 40-64.

**Comment 3**

*What are the limitations of the proposed study?*

Response: The Discussion now consolidates the principal limitations: only three principal study sources, heterogeneity among public studies, retrospective design, one small cohort, proxy tissue-composition measures, reference dependence, no linked NeoTRIP clinical outcomes in the available data, and only eight informative single-cell pairs.

Location in revised materials: Discussion, pp. 17-19, lines 368-425.

**Comment 4**

*The authors need to compare the proposed model with the existing state-of-the-art models.*

Response: A state-of-the-art prediction benchmark was not added because the manuscript evaluates a fixed candidate score rather than developing a new prediction model. The claim remains fixed-score association and cross-source reproducibility, distinct from deployable prediction.

Location in revised materials: Conclusions, p. 19, lines 426-432.

**Comment 5**

*What are the future directions of the proposed study?*

Response: We added a focused future-validation paragraph. Stronger validation requires genuinely independent study populations, a fixed single-sample scoring implementation or external reference distribution, prospectively specified preprocessing/analysis rules, more independent study sources, and prespecified treatment-interaction analysis for treatment-selection claims.

Location in revised materials: Future-validation statement, p. 18, lines 411-414.

**Comment 6**

*The authors need to revise the figures in 300 dpi for clear visualization.*

Response: All main figures were regenerated and are supplied as 600-dpi, RGB, LZW-compressed TIFFs meeting the journal size and width limits. Labels and panel structure were simplified, and all explicit figure text is 8 pt or larger.

Location in revised materials: Fig 1 caption, p. 12, lines 261-267; Fig 2 caption, p. 13, lines 283-287; Fig 3 caption, p. 15, lines 320-324; Fig 4 caption, p. 16, lines 339-341; Fig 5 caption, p. 16, lines 356-362.

# Reviewer #2

**Comment 1**

*GSE25055 and GSE25065 originated from the same parent study. Please justify treating them as separate cohorts in the primary meta-analysis and explain the effect on independence.*

Response: We retained the submitted four-accession analysis as primary to avoid changing the primary estimand after viewing results, but now make the dependence explicit. GSE25055 and GSE25065 retain separate accession-specific score references/intercepts; a main-text sensitivity combines them into one Hatzis/MD Anderson source effect using separate accession intercepts and one shared score slope, then pools that effect with GSE32646 and GSE163882. The three-source result is reported alongside, but is not called co-primary.

Location in revised materials: Study design/estimand, p. 4, lines 73-79; source-structure meta-analysis Methods, p. 8, lines 165-169; Results, p. 13, lines 278-292; Discussion, p. 18, lines 399-404; S1 Table S4.

**Comment 2**

*The score was calculated using within-cohort standardization. Please explain why this method was selected and discuss its limitations.*

Response: As noted above, the submitted reference-scope wording was corrected to match the executed analysis: target genes were standardized across the full processed matrix for each accession, followed by composite re-standardization in the analysis cohort. We added the mathematical definition and a single-sample RankScore sensitivity to assess dependence on cross-patient standardization.

Location in revised materials: Score construction, pp. 6-7, lines 121-150; RankScore Methods, pp. 8-9, lines 173-185; RankScore Results, p. 14, lines 296-302; Discussion, p. 17, lines 381-388; S2 PREPROCESSING_PROTOCOL.md, ANALYSIS_CHANGELOG.md, and code/02_rebuild_fixed_score_from_public_processed.py.

**Comment 3**

*The score correlated strongly with proliferation, while adjusted analyses were significant although the raw score was not. Please explain and provide correlations with the other adjustment variables.*

Response: The Results report the primary estimate first and distinguish direct adjustment, same-cohort residualization, and LOCO residualization as different estimands. Score–proliferation correlations are reported for every primary cohort, immune/stromal relationships are summarized across cohorts, and the full cohort-specific correlation matrix is shown in S1 Fig S3 with machine-readable tables in S2. Held-out LOCO residual correlations with proliferation, immune, and stromal variables quantify transport of the nuisance relationships.

Location in revised materials: Results, pp. 14-15, lines 303-328; Discussion, p. 18, lines 389-398; S1 Figs S3-S4 and S2 correlation tables.

**Comment 4**

*The LOCO residualization procedure is difficult to follow. Please provide a clearer step-by-step explanation, preferably with a schematic figure or workflow.*

Response: We rewrote LOCO as a stepwise training/held-out procedure: estimate nuisance slopes in the other cohorts with training-cohort intercepts, compute the training residual SD, apply the training-derived nuisance relation to the held-out cohort, express the held-out residual in training-SD units, fit a residual-only held-out pCR model, and pool fold effects. We also report held-out scale transfer and residual–background correlations in S1 Fig S4. The main Figure 1 shows the analysis hierarchy so LOCO is visually separated from the primary and direct adjusted models.

Location in revised materials: LOCO Methods, pp. 9-10, lines 195-208; Results, p. 15, lines 313-328; Fig 3 caption, p. 15, lines 320-324; S1 Fig S4.

**Comment 5**

*The authors set negative ComBat-adjusted values to zero before log transformation. Please provide a stronger justification for this approach.*

Response: As noted above, the submitted NeoTRIP description was corrected to match the executed raw-score analysis, which used the provider-supplied linear-scale matrix and all 401 profiles as the standardization reference. All 15 target-gene values were nonnegative in this matrix, so clipping is not relevant to the raw-score calculation. S2 documents the workflow and reported outputs.

Location in revised materials: NeoTRIP Methods, p. 10, lines 209-216; Results, p. 16, lines 335-345; Fig 4 caption, p. 16, lines 339-341; Discussion, p. 18, lines 393-398; S1 Table S9; S2 PREPROCESSING_PROTOCOL.md, ANALYSIS_CHANGELOG.md, and recovered_workflows/neotrip/.

**Comment 6**

*Please provide a clear table comparing patient characteristics, treatment regimens, sample processing, and pCR definitions across the primary cohorts.*

Response: We added a main-text cohort table reporting parent study/source, platform and pretreatment specimen, neoadjuvant treatment, retained pCR definition, and n/pCR. Unreported pathology details were left unspecified. Age, stage, and related patient-level variables were not consistently available in harmonizable form across all four public cohort inputs and were not imputed for cross-cohort comparison.

Location in revised materials: Cohort eligibility/characteristics Methods, pp. 4-5, lines 80-100; Table 1 and note, p. 5, lines 89-94; S1 Table S1; S2 INPUT_MANIFEST.tsv and PREPROCESSING_PROTOCOL.md.

**Comment 7**

*Please clearly report the definition of pCR used in each dataset and discuss differences.*

Response: The Methods and Table 1 now retain source-study definitions rather than retrospectively harmonizing them. GSE32646 required no residual invasive cancer in breast and axilla; GSE163882 used the pathology-report definition from the contributing institution; for GSE25055/GSE25065, the accessible source report did not support inferring a uniform nodal/DCIS rule. The limitation of non-uniform definitions is stated explicitly.

Location in revised materials: Cohort eligibility and source-defined pCR Methods, pp. 4-5, lines 80-94; Discussion limitations, p. 19, lines 415-425.

**Comment 8**

*Please provide more detail about how the final 15 genes were selected from the mitochondrial proteostasis/chaperone pathway and why other genes were excluded.*

Response: We now identify the parent 16-gene MitoCarta3.0 Chaperones pathway, list all 15 retained genes, specify the exact minimum 80% coverage scope across GPL96, GPL570, and the I-SPY2 expression platform, specify alias handling, and state that DNLZ was excluded because GPL96 lacked a reliable probe. GSE163882 was incorporated after confirming measurability of the 15 target genes. Equal positive weights and the no-cutpoint rule are also explicit.

Location in revised materials: Pathway definition and score construction, pp. 6-7, lines 121-150; S2 PREPROCESSING_PROTOCOL.md and INPUT_MANIFEST.tsv.

**Comment 9**

*The single-cell analysis includes few tumors. Please provide more details on malignant/non-malignant identification and pseudobulk analysis and emphasize the small sample size.*

Response: The analysis uses the processed single-cell inputs with source-provided patient field `orig.ident` and cell-type annotations; `Cancer Epithelial` is the malignant-epithelial label. Fig 5a retains patient×major-cell-type groups with at least 20 cells for display. Fig 5b eligibility requires one `Cancer Epithelial` row plus at least one non-cancer row with positive pooled counts. Eight of the 10 TNBC patients met that rule; CID3946 and CID44041 did not. Non-cancer counts are pooled within patient before CP10K, log1p, and 15-gene averaging. Patients—not cells—are the inferential units.

Location in revised materials: Single-cell Methods, pp. 10-11, lines 217-232; Results, p. 16, lines 346-362; Discussion, p. 18, lines 393-398 and pp. 19, lines 415-425; S1 Table S10.

**Comment 10**

*Please provide a more complete workflow from the original public datasets to the final results.*

Response: S2 now contains a public-input manifest, preprocessing protocol, patient-level derived analysis tables, platform-specific primary-score reconstruction and core-model workflows, source-structure meta-analysis, RankScore, single-cell analysis, figure regeneration, environment files, and hashes. The named public processed matrices and platform-annotation resources are listed in INPUT_MANIFEST.tsv.

Location in revised materials: Statistical software/reproducibility and Data and Code Availability, pp. 11-12, lines 233-252; S2 README.md, INPUT_MANIFEST.tsv, PREPROCESSING_PROTOCOL.md, code/, results/, SHA256SUMS.txt, and recovered_workflows/.

**Comment 11**

*Please state the specific knowledge gap more clearly in the Introduction.*

Response: The revised Introduction now frames two linked gaps: accession labels do not guarantee participant independence, and cohort-specific normalization can yield a reproducibly calculated but reference-dependent score. The study then evaluates a fixed score as a test case for limited cross-cohort association/reproducibility.

Location in revised materials: Introduction, p. 3, lines 47-64.

**Comment 12**

*The biological rationale for selecting a mitochondrial proteostasis-associated score in relation to TNBC response/pCR requires further explanation.*

Response: We expanded the biological bridge. The Introduction notes that chemotherapy-surviving TNBC models can show adaptive increases in mitochondrial oxidative phosphorylation and fusion, while MitoCarta defines mitochondrial chaperone/protein-quality-control functions. Mitochondrial proteostasis therefore provides a biologically plausible stress-response context for testing the fixed score.

Location in revised materials: Introduction, p. 3, lines 53-59; reference 4, p. 20.

**Comment 13**

*Please clarify whether the primary objective is to evaluate a specific mitochondrial gene score or demonstrate methodological challenges in public transcriptomic validation.*

Response: The primary objective is now stated explicitly as estimating the association between the fixed cohort-relative 15-gene score and source-study-defined pCR, with consistency assessed across study sources. Methodological issues such as overlap, reference dependence, and specimen context are interpreted as factors affecting that score evaluation rather than as a separate model-development objective.

Location in revised materials: Abstract, p. 2, lines 15-39; Introduction, p. 3, lines 60-64; primary estimand, p. 4, lines 73-79.

**Comment 14**

*Please provide more detail about how duplicate patients were identified and verified.*

Response: The matching process is now explicit. Matching was deterministic on source patient identifiers; receptor annotation, pCR concordance, and expression similarity were secondary verification checks and were not used to select matches. Three R1 repeats were collapsed, 58 IDs were present in GSE25055, 57 were strict TNBC in both accessions, M180 was receptor-discordant, and 10 had no matching identifier. The unmatched residual subset was not analyzed as a separate cohort.

Location in revised materials: Patient-overlap Methods, p. 6, lines 101-120; Results, p. 12, lines 254-277; Fig 1 caption, p. 12, lines 261-267; S1 Table S3; S2 data/overlap/ and protocol/01_Overlap_Audit_Prespecified.md.

**Comment 15**

*Please justify random-effects meta-analysis with few cohorts and provide sensitivity using alternative approaches/estimators.*

Response: We retained REML with modified Hartung-Knapp inference as the submitted primary procedure but now explicitly handle the tau²=0 boundary, report tau² and I² descriptively, and state that I²=0% is not proof of homogeneity. We added a fixed Paule–Mandel tau² sensitivity and the three-source sensitivity. The three-source interval is substantially wider and is reported alongside the primary result.

Location in revised materials: Meta-analysis/source-sensitivity Methods, p. 8, lines 160-169; Results, p. 13, lines 278-292; Discussion, p. 18, lines 399-404; S1 Table S4.

**Comment 16**

*Please strengthen the justification/limitations of within-cohort z-score normalization and, if possible, provide an alternative scoring sensitivity.*

Response: We now state that the cohort-relative score depends on accession-level and cohort-level reference distributions and therefore is not directly deployable for a new individual without a reference. A single fixed within-sample RankScore sensitivity was implemented using the same 15 genes and a fixed 12,151-gene background universe. The algorithm, universe, gene directions, cutoff rule, and cohort set were specified before evaluating the association.

Location in revised materials: Score construction, p. 7, lines 136-150; RankScore Methods, pp. 8-9, lines 173-185; Results, p. 14, lines 296-302; Discussion, p. 17, lines 381-388; S1 Table S5 and Fig S2; S2 code/04_rebuild_rank_score_from_public_processed.py.

**Comment 17**

*Please describe preprocessing by platform, including normalization, probe-to-gene mapping, multiple probes, and missing genes; a summary table would improve reproducibility.*

Response: We expanded both the manuscript and S2. Table 1 reports platform/specimen context. The score method specifies unambiguous symbol mapping, fixed aliases, within-sample median aggregation for multiple retained microarray probes and, for GSE163882, log2(TPM + 1) transformation followed by within-sample maximum aggregation of duplicate symbols, the platform-coverage rule, and the reference distributions. S2 INPUT_MANIFEST.tsv identifies the public processed expression matrices and platform-annotation resources by dataset and the packaged derived mapping tables. PREPROCESSING_PROTOCOL.md summarizes the score reference scopes and preprocessing provenance, while code/02_rebuild_fixed_score_from_public_processed.py implements the array probe-to-gene mapping, RNA-seq gene-level processing, alias handling, and score-reconstruction steps from the named public processed inputs.

Location in revised materials: Table 1 and note, p. 5, lines 89-94; pathway/score construction, pp. 6-7, lines 121-150; Data and Code Availability, pp. 11-12, lines 246-252; S2 INPUT_MANIFEST.tsv, PREPROCESSING_PROTOCOL.md, and code/02_rebuild_fixed_score_from_public_processed.py.

**Comment 18**

*The final paragraph of the Introduction contains substantial methodological detail. Please simplify it and focus on the research question and objectives.*

Response: We shortened the Introduction and moved technical implementation to the Methods. The final paragraph now states the fixed-score pCR question and the broad analysis components without enumerating model details.

Location in revised materials: Final Introduction paragraph, p. 3, lines 60-64.

# Reviewer #3

**Major comment 1**

*Clarify the precise estimand and intended claim; distinguish fixed-score association/cross-cohort reproducibility from clinical transportability or a deployable assay.*

Response: We agree and revised the framing throughout. The Methods define the primary estimand as the association between the fixed cohort-relative score and source-study-defined pCR, summarized across four accession-defined cohorts. Clinical deployment and treatment-effect prediction are outside that estimand. “Transportability” language implying clinical deployability was removed from the title and conclusions.

Location in revised materials: Abstract, p. 2, lines 15-39; Study design/estimand, p. 4, lines 66-79; Conclusions, p. 19, lines 426-432.

**Major comment 2**

*The effective number of independent study sources is very small; distinguish patients, accessions/cohorts, and study sources and avoid broad external-validation language.*

Response: We now report the hierarchy explicitly: 285 unique patients, four accession-defined cohorts, three principal study sources. GSE25055 and GSE25065 are treated as separate accessions in the primary analysis but one source in the source-structure sensitivity. The small number of sources is emphasized as a central limitation of precision and generalizability.

Location in revised materials: Abstract, p. 2, lines 20-37; Study design/estimand, p. 4, lines 73-79; Results, pp. 12-13, lines 268-292; Discussion, p. 18, lines 399-404; S1 Tables S1/S2/S4.

**Major comment 3**

*Provide stronger justification for random-effects meta-analysis with four cohorts, report tau², and do not interpret I²=0% as absence of heterogeneity.*

Response: The revised Methods compare the REML tau²=0 boundary with the numerical optimum, use mKH inference, report I² descriptively, and add Paule-Mandel tau² sensitivity. The primary four-cohort tau² is 0 after boundary comparison; the three-source REML tau² is 0.0201 and PM tau² is 0.00717. With only three to four analysis units, the manuscript reports effect estimates and confidence intervals without treating I²=0% as evidence of homogeneity.

Location in revised materials: Meta-analysis/source-sensitivity Methods, p. 8, lines 160-169; Results, p. 13, lines 278-292; Discussion, p. 18, lines 399-404; S1 Table S4.

**Major comment 4**

*The exploratory adjusted associations require especially cautious interpretation; make them subordinate to the primary null result and clarify LOCO.*

Response: We reorganized Results so the primary and source-level findings appear first. The adjusted/residual section labels the nominal findings as exploratory decomposition. LOCO is defined as a different estimand that tests whether nuisance relationships learned in other cohorts transport to the held-out cohort.

Location in revised materials: Primary/direct-adjustment Methods, p. 8, lines 151-172; LOCO Methods, pp. 9-10, lines 195-208; Table 2, p. 14, line 293; adjusted/LOCO Results, pp. 14-15, lines 303-328; Discussion, p. 18, lines 389-392.

**Major comment 5**

*The prior use of the same cohorts and outcomes introduces analytical history. Clarify when the 15-gene score was specified, prior related pCR analyses, changes after viewing pCR, and whether the internal record can be public.*

Response: The Methods now describe the 15-gene specification as fixed before the present score-specific pCR analysis, following related pCR analyses with a different mitochondrial gene set in overlapping cohorts. The Discussion explicitly acknowledges the resulting potential analytical flexibility; this retrospective specification was not a public preregistration or fully outcome-blinded development. The RankScore sensitivity was added during revision, with its implementation defined before evaluating its association with pCR. S2 includes the analytical-history record and revision protocol.

Location in revised materials: Pathway definition and score construction, pp. 6-7, lines 121-150; Discussion limitations, p. 19, lines 415-425; S2 ANALYTICAL_HISTORY.md, ANALYSIS_CHANGELOG.md, and protocol/.

**Major comment 6**

*Describe the patient-overlap matching procedure in greater detail, including identifiers, formatting, secondary confirmation, and uncertain matches.*

Response: We corrected and expanded the overlap audit. Matching is based on deterministic source patient identifiers; receptor annotations, pCR concordance, and expression similarity are secondary verification checks. Three repeat records were collapsed, 58 IDs were present in GSE25055, 57 were strict TNBC in both, M180 was receptor-discordant, and 10 were unmatched. The unmatched residual subset was not analyzed as a separate cohort. The full mapping/counterfactual tables are in S2/S1.

Location in revised materials: Patient-overlap Methods, p. 6, lines 101-120; Results, p. 12, lines 254-277; Fig 1 caption, p. 12, lines 261-267; S1 Table S3; S2 data/overlap/ and protocol/01_Overlap_Audit_Prespecified.md.

**Major comment 7**

*The cohort-relative standardization affects interpretation. Add a mathematical definition and, if possible, a cohort-independent/rank sensitivity.*

Response: We added the requested mathematical definition and corrected the submitted reference-scope wording as described above. The equation makes the accession-level and analysis-cohort reference distributions explicit. We also added one fixed single-sample RankScore sensitivity using the same 15 genes and a fixed 12,151-gene background universe.

Location in revised materials: Primary score construction, p. 7, lines 136-150; RankScore Methods, pp. 8-9, lines 173-185; Results, p. 14, lines 296-302; Discussion, p. 17, lines 381-388; S2 PREPROCESSING_PROTOCOL.md, ANALYSIS_CHANGELOG.md, code/02_rebuild_fixed_score_from_public_processed.py, and code/04_rebuild_rank_score_from_public_processed.py.

**Major comment 8**

*NeoTRIP preprocessing requires a non-clipping sensitivity or stronger justification.*

Response: The submitted NeoTRIP description was corrected as described above. The executed raw-score analysis used the provider-supplied linear-scale matrix and all 401 profiles as the standardization reference; all 15 target-gene values were nonnegative. The background-feature decompositions remain contextual rather than a primary mechanistic test.

Location in revised materials: NeoTRIP Methods, p. 10, lines 209-216; Results, p. 16, lines 335-345; Fig 4 caption, p. 16, lines 339-341; Discussion, p. 18, lines 393-398; S1 Table S9; S2 PREPROCESSING_PROTOCOL.md, ANALYSIS_CHANGELOG.md, and recovered_workflows/neotrip/.

**Major comment 9**

*The single-cell analysis is informative but underpowered; eight paired patients should be treated as hypothesis-generating.*

Response: We treat the single-cell analysis as contextual and patient-level. The analysis uses the 10-TNBC subset of GSE176078 and source-provided `orig.ident` and cell-type annotations; `Cancer Epithelial` is the malignant epithelial label used here. Fig 5b eligibility requires one `Cancer Epithelial` row plus non-cancer rows with positive pooled counts. Eight patients met that rule; CID3946 and CID44041 did not. S2 includes the reduced cell-level inputs and executable workflow, including the cycling-state sensitivity.

Location in revised materials: Single-cell Methods, pp. 10-11, lines 217-232; Results, p. 16, lines 346-362; Discussion, p. 18, lines 393-398 and pp. 19, lines 415-425; S1 Table S10; S2 recovered_workflows/single_cell/.

**Major comment 10**

*Distinguish absence of evidence from evidence of absence.*

Response: The Abstract, Discussion, and Conclusion now state the result directly: neither fixed implementation showed a reproducible cross-cohort association, and the study-source estimates remained imprecise. The wide intervals indicate limited precision around the effect estimate.

Location in revised materials: Abstract, p. 2, lines 27-37; Results, pp. 13-14, lines 278-302; Discussion, pp. 17-18, lines 368-404; Conclusions, p. 19, lines 426-432.

**Major comment 11**

*Keep I-SPY2 clearly separate from the primary TNBC analysis and avoid implying that it validates the score.*

Response: I-SPY2 remains contextual evidence, not a strict-TNBC primary cohort. The patient-level I-SPY2 analysis includes treatment-arm-adjusted, arm+MammaPrint, and score-by-arm interaction models. Because I-SPY2 uses HR-negative/HER2-negative eligibility and a multi-arm treatment structure, those analyses remain outside the primary four-cohort meta-analysis.

Location in revised materials: I-SPY2 source boundary, p. 5, lines 95-100; Results, p. 17, lines 363-367; Discussion, p. 18, lines 405-410; S1 Table S11.

**Major comment 12**

*Retain the distinction between response association and treatment prediction.*

Response: The manuscript distinguishes response association from treatment-effect prediction. Treatment-selection claims require prespecified, reproducible score-by-treatment interaction evidence.

Location in revised materials: I-SPY2 Discussion, p. 18, lines 405-410; future-validation statement, p. 18, lines 411-414.

**Data availability and reproducibility**

*Provide complete preprocessing scripts or a sufficiently detailed step-by-step preprocessing protocol, and ensure S2 contains all variables needed to reproduce every primary estimate.*

Response: S2 now addresses this requirement. Core estimates can be re-fitted from the 285-patient derived table; score construction is traceable to explicitly named public processed matrices and platform annotations; the fixed RankScore workflow enforces the 12,151-gene universe by gene identity; and the source-level LOCO diagnostic is executable from the 285-patient table. S2 also supplies executable refits for patient-level I-SPY2 models and a reduced-input single-cell workflow. For NeoTRIP, S2 provides derived tables, outputs, historical generator code, and archived checks; the generator requires the original project inputs and path configuration.

Location in revised materials: Statistical software/reproducibility and Data and Code Availability, pp. 11-12, lines 233-252; S2 README.md, INPUT_MANIFEST.tsv, PREPROCESSING_PROTOCOL.md, code/, results/, SHA256SUMS.txt, and recovered_workflows/.

**Statistical reporting**

*Report cohort events/non-events and model n; tau²; correlation confidence-interval methods; small-sample corrections; the complete exploratory analysis set/multiplicity; and acknowledge limited power.*

Response: These items are documented in the revision. Cohort/model n and pCR events are reported in the main Methods and S1 Table S2; tau² is reported for primary and sensitivity meta-analyses; S1 Supplementary Methods specifies Fisher-z correlation intervals and Fisher-z random-effects pooling; it also states that ordinary binomial GLM/Wald intervals were used, no Firth penalty was applied, and all retained core fits converged without complete separation. S1/S2 inventory the exploratory families and FDR procedures. The Discussion emphasizes the small number of independent study sources and the resulting imprecision.

Location in revised materials: Results, pp. 13-16, lines 278-362; Discussion, pp. 18-19, lines 399-425; S1 Supplementary Methods and Tables S2, S4, S12; S2 results/.

**Presentation and language**

*Shorten complex methodological sentences and ensure consistent terminology for cohort, accession, study source, patient, record, and sample.*

Response: The Methods were reorganized into topic-specific subsections, and terminology was standardized. “Patient” refers to the biological participant, “record/aliquot” to observations that may duplicate a patient, “accession-defined cohort” to the analytic dataset, and “study source” to the parent population/source. These terms are used consistently throughout the revised manuscript.

Location in revised materials: Reorganized Methods, pp. 4-12, lines 65-252; patient/accession/source terminology in overlap and source-structure sections, pp. 6-8, lines 101-168 and pp. 12-13, lines 254-292.

**Minor comment 1**

*Define LOCO and computational abbreviations at first use.*

Response: LOCO is now introduced as leave-one-cohort-out before abbreviation, and key computational terms are expanded or described in context.

Location in revised materials: LOCO Methods, pp. 9-10, lines 195-208; Results, p. 15, lines 313-328; S2 code/08_rebuild_leave_one_source_out.py.

**Minor comment 2**

*Distinguish patients, samples, aliquots, and records.*

Response: The overlap/aliquot sections now use these terms explicitly and separately; all clinical analyses use one row per patient after the GSE163882 aliquot collapse.

Location in revised materials: overlap Methods, p. 6, lines 101-120; overlap Results, p. 12, lines 254-277.

**Minor comment 3**

*Provide a transparent flow diagram showing records, overlap, aliquot handling, eligibility, and final analysis numbers.*

Response: Figure 1 was redesigned as a two-path flow diagram separating the primary candidate/deduplication path from the GSE20194 overlap audit and descriptive counterfactual.

Location in revised materials: Fig 1 caption, p. 12, lines 261-267; S1 Table S3.

**Minor comment 4**

*Explain the treatment of unmatched GSE20194 records and why the entire accession was excluded.*

Response: The revised audit identifies 10 GSE20194 patients with no matching GSE25055 identifier. Because participant reuse was extensive, GSE20194 was excluded as an independent primary cohort; the unmatched residual subset was not analyzed as a separate cohort.

Location in revised materials: overlap Methods, p. 6, lines 101-120; overlap Results, p. 12, lines 254-277.

**Minor comment 5**

*Clarify that this is a computational secondary analysis and not wet-laboratory experimental validation.*

Response: The Study Design section identifies the work as a retrospective secondary analysis of publicly accessible, de-identified transcriptomic and clinical data. The manuscript does not present the study as a new experimental or wet-laboratory validation.

Location in revised materials: Study design and reporting, p. 4, lines 66-72; Statistical software/reproducibility, p. 11, lines 233-242.

**Minor comment 6**

*Ensure all substantive methodological/statistical decisions remain attributable to the investigators rather than AI-assisted suggestions.*

Response: All substantive methodological and statistical decisions were made by the investigators. OpenAI ChatGPT was used for language editing, code review, and consistency checking. The authors are responsible for all data, analyses, results, and manuscript content.

Location in revised materials: Use of generative artificial intelligence, p. 11, lines 243-245.

# Reviewer #4

**Overall recommendation**

*Reviewer #4 recommended acceptance and provided no additional requested changes.*

Response: We thank Reviewer 4 for the free-text recommendation to accept the manuscript and note that no specific revision requests were provided. We addressed the Editor and other reviewers in detail.

Location in revised materials: clean revised manuscript with final continuous line numbering; supporting files as cited in this response.

# Files supplied with the revision

- PONE-D-26-36973_Revised_Manuscript_Clean.docx
- PONE-D-26-36973_Revised_Manuscript_Tracked.docx
- Response_to_Reviewers.docx
- S1_Appendix_Revised.pdf
- S2_File_Data_Code.zip
- Figures/Fig1.tif through Figures/Fig5.tif (600-dpi RGB/LZW TIFF)
- Figure source-data tables: included within S2 File under results/figure_source/
