# Supplementary material for: Patient overlap, cohort-relative scaling, and biological context in cross-cohort evaluation of a mitochondrial proteostasis-associated score in triple-negative breast cancer
Source: PLoS One. 2026 Oct 9;21(10):e0355652. doi: 10.1371/journal.pone.0355652 (PMC13652229; doi:10.1371/journal.pone.0355652)
Supplement: Peer review history

# Peer review: Decision Letter 0

**DOI:** 10.1371/journal.pone.0355652.r001

**Authors:** UDAYAN BHATTACHARYA

14 Sep 2026

Dear Dr.  Weng,

Thank you for submitting your manuscript to PLOS One. After careful consideration, we feel that it has merit but does not fully meet PLOS One’s publication criteria as it currently stands. Therefore, we invite you to submit a revised version of the manuscript that addresses the points raised during the review process.

Please submit your revised manuscript by Nov 13 2026 11:59PM If you will need more time than this to complete your revisions, please reply to this message or contact the journal office at plosone@plos.org. When you're ready to submit your revision, log on to https://www.editorialmanager.com/pone/ and select the 'Submissions Needing Revision' folder to locate your manuscript file.

- A letter that responds to each point raised by the academic editor and reviewer(s). You should upload this letter as a separate file labeled 'Response to Reviewers'.
- A marked-up copy of your manuscript that highlights changes made to the original version. You should upload this as a separate file labeled 'Revised Manuscript with Track Changes'.
- An unmarked version of your revised paper without tracked changes. You should upload this as a separate file labeled 'Manuscript'.

If you would like to make changes to your financial disclosure, please include your updated statement in your cover letter. Guidelines for resubmitting your figure files are available below the reviewer comments at the end of this letter.

If applicable, we recommend that you deposit your laboratory protocols in protocols.io to enhance the reproducibility of your results. Protocols.io assigns your protocol its own identifier (DOI) so that it can be cited independently in the future. For instructions see: https://journals.plos.org/plosone/s/submission-guidelines#loc-laboratory-protocols. Additionally, PLOS One offers an option for publishing peer-reviewed Lab Protocol articles, which describe protocols hosted on protocols.io. Read more information on sharing protocols at https://plos.org/protocols?utm_medium=editorial-email&utm_source=authorletters&utm_campaign=protocols.

As the corresponding author, your ORCID iD is verified in the submission system and will appear in the published article. PLOS supports the use of ORCID, and we encourage all coauthors to register for an ORCID iD and use it as well. Please encourage your coauthors to verify their ORCID iD within the submission system before final acceptance, as unverified ORCID iDs will not appear in the published article. *Only* the individual author can complete the verification step; PLOS staff *cannot* verify ORCID iDs on behalf of authors.

We look forward to receiving your revised manuscript.

Kind regards,

UDAYAN BHATTACHARYA

Academic Editor

PLOS One

Journal Requirements:

When submitting your revision, we need you to address these additional requirements.

1.Please ensure that your manuscript meets PLOS ONE's style requirements, including those for file naming. The PLOS ONE style templates can be found at

https://journals.plos.org/plosone/s/file?id=wjVg/PLOSOne_formatting_sample_main_body.pdf and

https://journals.plos.org/plosone/s/file?id=ba62/PLOSOne_formatting_sample_title_authors_affiliations.pdf

2. Please note that your Data Availability Statement is currently missing the repository name and/or the DOI/accession number of each dataset OR a direct link to access each database. If your manuscript is accepted for publication, you will be asked to provide these details on a very short timeline. We therefore suggest that you provide this information now, though we will not hold up the peer review process if you are unable.

3. Please upload a new copy of Figure 1 to 5 as the detail is not clear. Please follow the link for more information:  https://journals.plos.org/plosone/s/figures.

4. If the reviewer comments include a recommendation to cite specific previously published works, please review and evaluate these publications to determine whether they are relevant and should be cited. There is no requirement to cite these works unless the editor has indicated otherwise.

Additional Editor Comments:

Dear Dr. Weng,

Thank you for submitting your manuscript, "Patient overlap, cohort-relative scaling, and biological context in cross-cohort validation of a mitochondrial proteostasis-associated score in triple-negative breast cancer" (PONE-D-26-36973), to PLOS ONE.

Your manuscript has been evaluated by four expert reviewers. While they find the work promising, significant concerns have been raised regarding the patient overlap across cohorts, scaling methodology, and biological contextualization. Based on these evaluations, we invite you to submit a major revision of your manuscript.

Please address all points raised by the reviewers in a comprehensive, point-by-point response and update the manuscript text and figures accordingly.

Key Requirements for Resubmission:

Response to Reviewers: Provide a detailed point-by-point response addressing every comment. Indicate the exact changes made and specify page and line numbers in the revised manuscript where edits were incorporated.

Manuscript Files: Upload both a clean version of the revised manuscript and a marked-up copy highlighting all modifications (via track changes or text highlighting).

Data and Code Integrity: Ensure all underlying data, cohort definitions, and scaling code are clearly documented and accessible in accordance with PLOS ONE policies.

Please submit your revised manuscript within 45 days. If additional time is needed to complete the required analyses or revisions, please contact the journal office to request an extension.

We look forward to receiving your revised submission.

Sincerely,

Udayan Bhattacharya

Academic Editor

[Note: HTML markup is below. Please do not edit.]

Reviewer's Responses to Questions

**Comments to the Author**

1. Is the manuscript technically sound, and do the data support the conclusions?

Reviewer #1: Yes

Reviewer #2: Partly

Reviewer #3: Partly

Reviewer #4: No

**********

2. Has the statistical analysis been performed appropriately and rigorously?

Reviewer #1: Yes

Reviewer #2: Yes

Reviewer #3: Yes

Reviewer #4: No

**********

3. Have the authors made all data underlying the findings in their manuscript fully available?

The PLOS Data policy

Reviewer #1: Yes

Reviewer #2: Yes

Reviewer #3: Yes

Reviewer #4: Yes

**********

4. Is the manuscript presented in an intelligible fashion and written in standard English?

Reviewer #1: Yes

Reviewer #2: Yes

Reviewer #3: Yes

Reviewer #4: Yes

**********

Reviewer #1: 1. In the abstract, the authors need to mention the improvement of the proposed model as compared to the existing models.

2. At the introduction , the key contributions of the proposed model should be concise and clearly provided.

3. What are the limitations of the proposed study?

4. The authors need to compare the proposed model with the existing state-of-the-art models

5. What are the future directions of the proposed study?

6. The authors need to revise the figures in 300 dpi for clear visualization.

Reviewer #2: Reviewer Comments

Overall recommendations: Major Revision

This manuscript addresses an important issue in transcriptomic biomarker validation, particularly patient overlap between public datasets and the influence of cohort-specific normalization and biological factors. The study is interesting; however, several methodological and interpretational issues should be clarified before the manuscript can be considered for publication.

Comment 1: The manuscript states that GSE25055 and GSE25065 originated from the same parent study. Please provide more justification for treating them as separate cohorts in the primary meta-analysis. It would also be helpful to explain whether this shared origin could affect the independence of the results.

Comment 2: The score was calculated using within-cohort standardization. Please explain more clearly why this normalization method was selected and discuss its limitations.

Comment 3: The score showed a strong correlation with proliferation. However, the raw score was not associated with pCR, while some adjusted analyses showed a significant association. Please explain this result more clearly. It would also be helpful to provide information about the correlation between the score and the other variables used in the adjustment models.

Comment 4: The leave-one-cohort-out (LOCO) residualization procedure is central to the study. However, the methodology is difficult to follow. Please provide a clearer step-by-step explanation of the procedure, preferably with a schematic figure or workflow.

Comment 5: The authors set negative ComBat-adjusted values to zero before log transformation. Please provide a stronger justification for this approach.

Comment 6: The included datasets may differ in patient characteristics, treatment regimens, sample processing, and definitions of pCR. Please provide a clear table comparing these characteristics across the primary cohorts. This will help readers understand the possible sources of differences between cohorts.

Comment 7: Please clearly report the definition of pathological complete response used in each dataset. Were the same criteria used in all cohorts? If the definitions were different, please discuss whether this could affect the meta-analysis.

Comment 8: Please provide more detail about how the final 15 genes were selected from the

mitochondrial proteostasis/chaperone pathway. Were all genes from the original pathway

included? If not, please clearly explain the selection criteria and the reason for excluding other

genes.

Comment 9: The single-cell analysis is interesting, but it includes a limited number of tumors.

Please provide more details about how malignant and non-malignant cells were identified and how

the pseudobulk analysis was performed. The limitations of the small sample size should also be

emphasized.

Comment 10: The authors provide scripts and derived data, which is appreciated. However, the

manuscript states that not all upstream preprocessing steps can be fully reproduced. Please

consider providing a more complete workflow from the original public datasets to the final results.

Comment 11: The Introduction provides a useful overview of the challenges of cross-cohort

transcriptomic biomarker validation. However, the specific knowledge gap addressed by this study

should be stated more clearly.

Comment 12: The biological rationale for selecting a mitochondrial proteostasis-associated gene

score in relation to TNBC treatment response and pCR requires further explanation.

Comment 13: The manuscript should clarify whether the primary objective is to evaluate a

specific mitochondrial gene score or to demonstrate methodological challenges in public

transcriptomic biomarker validation.

Comment 14: The identification of overlapping patients is an important aspect of this study.

However, please provide more detail about how duplicate patients were identified and verified.

Please clarify which variables or identifiers were used and how the authors confirmed that the

identified samples represented the same patients rather than merely similar clinical characteristics.

Comment 15: Please provide additional justification for the selected random-effects meta-analysis

approach. Because the primary analysis includes a limited number of cohorts,

heterogeneity estimates may be unstable. It would be helpful to provide sensitivity analyses using

alternative meta-analysis approaches or estimators.

Comment 16: Please provide a stronger justification for the use of within-cohort z-score

normalization. Since this approach depends on the distribution of the cohort, the resulting score

may not be directly applicable to an individual patient or an independent dataset without

recalculation. Please discuss this limitation clearly and, if possible, provide sensitivity analyses

using an alternative scoring method.

Comment 17: The study combines datasets generated using different gene-expression platforms.

Please provide a clearer description of the preprocessing steps applied to each dataset, including

normalization, probe-to-gene mapping, handling of multiple probes representing the same gene,

and treatment of missing genes. A summary table describing preprocessing for each dataset would

improve reproducibility.

Comment 18: The final paragraph of introduction contains substantial methodological detail.

Consider simplifying it and focusing more clearly on the research question and main objectives.

Reviewer #3: Overall assessment

This manuscript addresses an important and often underappreciated problem in secondary analyses of public transcriptomic datasets: the assumption that different accession numbers necessarily represent independent patient populations and that a molecular score that can be calculated across platforms is automatically transportable. The authors have undertaken a thoughtful multi-cohort analysis of a fixed 15-gene mitochondrial proteostasis-associated score in triple-negative breast cancer (TNBC), with particular attention to patient overlap, cohort-relative normalization, proliferation, tissue composition, longitudinal treatment-related changes, and single-cell localization.

The manuscript has several strengths. These include explicit patient-level deduplication, separation of primary from exploratory analyses, use of leave-one-cohort-out residualization, consideration of study-source dependence, transparent reporting of uncertainty, and an appropriately cautious interpretation of the principal null finding. The authors also acknowledge important limitations rather than overstating the clinical implications. For example, the primary analysis included 285 patients from four cohorts and found no significant association between the raw score and pCR (OR 0.958, 95% CI 0.639–1.437, P=0.760), while the exploratory adjusted associations were not reproduced under held-out residualization. These findings are appropriately presented as evidence against reproducible cross-cohort transportability rather than definitive proof of absence of biological effect.

Nevertheless, I recommend major revision. My principal concerns relate to the distinction between testing a fixed biological score and developing/validating a clinically transportable biomarker, the limited number of independent study sources, potential analytical dependence between cohorts, the interpretation of exploratory adjusted analyses, and the reproducibility of the upstream transcriptomic preprocessing.

Major comments

1. Clarify the precise estimand and intended claim of the study

The manuscript alternates between evaluating a “fixed score,” assessing “cross-cohort transportability,” and discussing implications for clinical biomarker implementation. These are related but distinct objectives. The authors should state explicitly at the beginning of the Methods what constitutes the primary estimand: association between the score and pCR, reproducibility of that association across independent cohorts, or transportability of the score as a clinical prediction tool.

This distinction is particularly important because the score is calculated after within-cohort z-standardization. The authors correctly acknowledge that such a score cannot be applied to an individual new patient without a reference distribution. Therefore, the present analysis does not actually validate a deployable clinical assay.

I recommend revising the terminology throughout the manuscript so that “transportability” does not imply clinical deployability. A more precise formulation would be that the study evaluates cross-cohort reproducibility of a cohort-relative transcriptomic score, rather than clinical transportability per se.

2. The effective number of independent study sources is very small

Although 285 patients are included, the primary analysis comprises only four cohorts originating from three principal study sources. Furthermore, GSE25055 and GSE25065 originate from the same parent study. Thus, the nominal sample size should not be allowed to obscure the relatively small number of independent sources informing the meta-analytic inference. The authors acknowledge this limitation, but its implications for statistical power and generalizability deserve greater emphasis.

Please provide, preferably in the main manuscript or supplementary material, a clear distinction between:

number of patients;

number of accession datasets;

number of independent cohorts;

number of independent study populations/sources.

I also recommend avoiding language suggesting that the analysis constitutes broad external validation when only three principal study sources are represented.

3. Provide stronger justification for the random-effects meta-analysis with only four primary cohorts

The use of REML with modified Hartung–Knapp inference is defensible, and it is preferable to relying solely on conventional inverse-variance methods. However, with only four cohorts, estimation of between-cohort heterogeneity is intrinsically imprecise. The reported I²=0% for the primary analysis should therefore not be interpreted as evidence that heterogeneity is absent.

The authors appropriately used random-slope sensitivity analyses and explicitly noted weak identification of variance components. Nevertheless, I suggest reporting the estimated between-study variance (τ²), where meaningful, alongside I² and explaining the limitations of interpreting heterogeneity statistics with four studies.

The manuscript should also emphasize that a non-significant heterogeneity statistic does not establish true homogeneity.

4. The exploratory adjusted associations require particularly cautious interpretation

The score was not associated with pCR in the primary model, but became nominally significant after adjustment for proliferation (OR 0.553, P=0.043), after adjustment for proliferation, immune and stromal scores (OR 0.473, P=0.032), and following post hoc within-cohort residualization (OR 0.633, P=0.042). None was reproduced in the leave-one-cohort-out analysis (OR 0.790, 95% CI 0.439–1.420, P=0.290).

The authors appropriately call these analyses exploratory, but the presentation could nevertheless lead readers to focus on the nominally significant adjusted associations.

I recommend restructuring the Results and Discussion so that these findings are clearly subordinate to the primary null analysis. In particular, explain that residualization performed using the same cohorts used for outcome evaluation can induce optimistic or dataset-specific associations, whereas the LOCO procedure provides a more stringent test of generalizability.

The manuscript makes this point well in the Discussion, but it should be made even more explicit in the Results.

5. The prior use of the same cohorts and outcomes introduces an important form of analytical history

The authors state that the 15-gene score was fixed before testing against pCR, but the same public cohorts and pCR outcomes had previously been examined in a separate analysis involving a different mitochondrial gene set. The authors also acknowledge that the current score was not prospectively preregistered or developed under complete outcome blinding.

This is an important limitation and should be more prominently discussed as a potential source of analytical flexibility, even though no outcome-adaptive gene selection or weighting was performed in the present analysis.

Please clarify:

when the 15-gene score was first specified;

whether the investigators had examined pCR associations with related mitochondrial gene sets before fixing this score;

whether any aspects of the present analysis plan were modified after viewing pCR results;

whether the internal retrospective record can be made publicly accessible.

The distinction between prespecified and prospectively registered should be maintained carefully.

6. Patient-overlap analysis is a major strength, but the matching procedure should be described in greater detail

The identification of 55 matching patients between GSE20194 and GSE25055 is important and convincingly demonstrates why accession-level independence cannot simply be assumed. The authors report concordant outcomes and a Pearson correlation of 0.963 for independently calculated score values.

However, the manuscript should provide more detail about the matching process. Specifically, please describe:

which identifiers were used;

how identifier formatting differences were resolved;

whether matching was deterministic or involved manual review;

whether clinical variables or specimen characteristics were used as secondary confirmation;

how uncertain or partial matches were handled.

This would substantially strengthen reproducibility of one of the manuscript's central methodological contributions.

7. The cohort-relative standardization fundamentally affects interpretation of the score

Within each cohort, each gene was z-standardized, the 15 genes were averaged, and the composite was again standardized. This approach makes cross-platform computation possible but also means that the score is inherently dependent on the distribution of the cohort.

The manuscript recognizes this limitation, but I recommend adding a simple mathematical definition of the score and explicitly illustrating how the same biological sample could receive different values depending on the reference cohort. This would make the central methodological issue much clearer to readers.

If possible, a sensitivity analysis using a cohort-independent or rank-based scoring approach would substantially strengthen the manuscript. If such an analysis is outside the scope of the current work, the authors should explain why and identify it as a priority for future validation.

8. The NeoTRIP preprocessing requires additional sensitivity analysis or stronger justification

The authors state that negative ComBat-adjusted values were set to zero before log2(x+1) transformation and explicitly acknowledge that the transformation was not compared with a non-clipping alternative.

This is potentially important because the longitudinal score changes are one of the principal pieces of biological evidence supporting the contextual interpretation. The large decrease in the raw score and proliferation after treatment is striking, but if the preprocessing transformation materially affects these values, the biological interpretation should remain provisional.

I recommend, if technically feasible, repeating the NeoTRIP analysis using at least one alternative defensible preprocessing approach without clipping negative values, and comparing the direction and magnitude of the conclusions. If this cannot be done because of limitations in the public data, the authors should expand the limitation statement and avoid language suggesting that the longitudinal findings independently demonstrate biological suppression of mitochondrial proteostasis.

9. The single-cell analysis is informative but substantially underpowered for biological localization

The single-cell analysis includes 42,512 cells from only 10 TNBC tumors, with eight informative paired malignant-versus-noncancer comparisons. The use of patients rather than individual cells as the inferential unit is appropriate and avoids pseudoreplication.

However, P=0.0078 from eight paired patients should not be interpreted as strong evidence of general tumor-cell specificity. The authors appropriately conclude that the score is enriched in malignant epithelium but is not tumor-cell-specific. I recommend explicitly describing this analysis as a small patient-level localization analysis and emphasizing that it is hypothesis-generating.

10. The conclusion should distinguish absence of evidence from evidence of absence

The primary OR of 0.958 has a relatively wide 95% CI (0.639–1.437). Thus, although the point estimate is close to the null, the confidence interval remains compatible with clinically or biologically meaningful effects in either direction. The authors correctly state this in the Discussion.

I recommend carrying this caution consistently into the Abstract and Conclusion. The preferred interpretation is that the study did not demonstrate a reproducible association under the specified scoring and cohort structure, rather than that the mitochondrial score has no relationship with pCR.

11. The I-SPY2 analysis should remain clearly separated from the primary TNBC analysis

The I-SPY2 population is HR-negative/HER2-negative rather than strictly defined TNBC, and the treatment structure differs from the primary cohorts. The manuscript appropriately keeps it outside the primary meta-analysis.

This is appropriate. However, because the I-SPY2 result is statistically significant (OR 0.716, P=0.005), it may attract disproportionate attention. I recommend making its status as contextual, non-primary evidence particularly prominent in the Abstract, Results, figure legends, and Discussion.

The authors should avoid language that could imply that the I-SPY2 result validates the score after the primary analysis was null.

12. The treatment-predictive versus prognostic/predictive distinction should be retained

The authors correctly state that an association with response is different from treatment prediction and that the absence of a significant score-by-treatment interaction does not support treatment selection.

This is an important strength. I recommend retaining this distinction prominently because readers may otherwise interpret the I-SPY2 association as evidence for treatment selection.

Data availability and reproducibility

The data availability statement is substantially stronger than is typical for secondary analyses. The manuscript identifies the GEO accessions and states that S2 File contains patient-level overlap information, derived patient-level data, model outputs, figure source data, software-environment files, and reproduction/verification scripts.

I therefore consider the data underlying the principal reported findings to be sufficiently available in principle. However, the authors should address one important reproducibility limitation: the manuscript explicitly states that the raw expression preprocessing is not fully reconstructed in the archive.

For maximum reproducibility, please provide either:

complete preprocessing scripts; or

a sufficiently detailed step-by-step preprocessing protocol, including probe selection, gene mapping, transformations, handling of missing/negative values, normalization, and platform-specific processing.

The authors should also ensure that S2 File contains all variables necessary to reproduce every primary estimate without requiring undocumented manual processing.

Statistical reporting

The statistical reporting is generally strong. The authors provide effect estimates, 95% confidence intervals and P values rather than relying only on significance testing. The linearity assessment, study-source sensitivity analysis, LOCO residualization, multiplicity correction, and random-slope sensitivity analyses are commendable.

Nevertheless, the authors should consider reporting:

the number of events and non-events in every cohort;

cohort-specific sample sizes used in each adjusted model;

τ² where applicable;

exact methods used to calculate confidence intervals for the meta-analytic correlations;

whether any small-sample corrections were considered for cohort-specific logistic regression;

the complete set of exploratory analyses performed, preferably in supplementary material, to make the analytical multiplicity transparent.

The absence of a prospective sample-size calculation is understandable for a retrospective analysis using all eligible public data, and I would not consider this alone grounds for rejection. However, the manuscript should explicitly acknowledge that the study may have limited power to exclude moderate effects because only four primary cohorts were available.

Presentation and language

The manuscript is generally well written, technically literate, and easy to follow. The narrative is coherent and the authors distinguish primary analyses from exploratory analyses better than many biomarker studies.

Some methodological sentences are lengthy and contain multiple concepts. Shortening several of these sentences would improve readability for readers outside computational oncology. In particular, the Methods sections dealing with score construction, residualization, and NeoTRIP preprocessing could benefit from concise subheadings and/or schematic equations.

The manuscript should also undergo a final consistency check for terminology such as “cohort,” “accession,” “study source,” “patient,” “record,” and “sample,” because these terms have different meanings in this study.

Additional minor comments

Please ensure that every abbreviation is defined at first use in the main manuscript, including LOCO and any computational terminology that may be unfamiliar to the broader PLOS ONE readership.

The manuscript should clearly distinguish “patients,” “samples,” “aliquots,” and “records” throughout. This is particularly important in the patient-overlap analysis.

Please provide a transparent flow diagram showing the initial number of records, exclusions because of patient overlap, duplicate aliquot handling, eligibility exclusions, and final numbers contributing to each analysis.

The treatment of the two unmatched GSE20194 records should be explained more explicitly. Although excluding the entire accession is defensible, readers should understand why those records could not be independently verified.

The statement that no wet-laboratory experiments were performed is appropriate, but it would be useful to make clear that the manuscript represents a computational secondary analysis rather than an experimental validation study.

The AI-use statement is transparent and appropriate. Nevertheless, the authors should ensure that all substantive methodological and statistical decisions remain demonstrably attributable to the investigators rather than to AI-assisted suggestions.

Recommendation

Overall, I consider this a potentially valuable methodological and biological contribution. The central message—that public transcriptomic biomarker validation can be misleading when patient overlap, cohort-relative normalization, proliferation, and specimen composition are not adequately considered—is important and is generally supported by the presented analyses.

However, the manuscript should undergo major revision before publication. The revisions should particularly strengthen the definition of the primary estimand, clarify the implications of having only three principal study sources/four primary cohorts, further justify the meta-analytic strategy, distinguish cross-cohort reproducibility from clinical transportability, provide greater transparency concerning patient matching and prior analytical use of the datasets, and address the sensitivity of the NeoTRIP findings to preprocessing.

Importantly, the authors should preserve the cautious tone already present in the Discussion and Conclusion. The strongest contribution of this work is not demonstrating that the 15-gene score has absolutely no biological relationship with TNBC response, but demonstrating that its apparent association is not robustly transportable under the evaluated cohort-relative framework and may be substantially influenced by proliferation and specimen context. That conclusion is both scientifically defensible and potentially useful to the field.

Reviewer #4: I recommend acceptance of this manuscript. The study is carefully conducted, transparent, and makes a useful contribution by highlighting important practical issues in the validation of transcriptomic biomarkers from public cohorts.

**********

what does this mean?). If published, this will include your full peer review and any attached files.

If you choose “no”, your identity will remain anonymous but your review may still be made public.

**Do you want your identity to be public for this peer review?** For information about this choice, including consent withdrawal, please see our Privacy Policy

Reviewer #1: No

Reviewer #2: No

Reviewer #3: **Yes:**  Saheed Ayodeji Adekola

Reviewer #4: No

**********

[NOTE: If reviewer comments were submitted as an attachment file, they will be attached to this email and accessible via the submission site. Please log into your account, locate the manuscript record, and check for the action link "View Attachments". If this link does not appear, there are no attachment files.]

To ensure your figures meet our technical requirements, please review our figure guidelines: https://journals.plos.org/plosone/s/figures

You may also use PLOS’s free figure tool, NAAS, to help you prepare publication quality figures: https://journals.plos.org/plosone/s/figures#loc-tools-for-figure-preparation.

NAAS will assess whether your figures meet our technical requirements by comparing each figure against our figure specifications.

Attachment Submitted filename: Reviewer Comments.pdf

# Author comment: Author response to Decision Letter 1

**DOI:** 10.1371/journal.pone.0355652.r002

20 Sep 2026

Academic Editor and journal requirements

Journal requirement 1

Please ensure that your manuscript meets PLOS ONE style requirements, including file naming.

Response: We reformatted and renamed the revision package consistently and provide separate clean and tracked manuscripts, S1 Appendix, S2 File, individual high-resolution figures, and the response letter. The manuscript title was also narrowed from “validation/transportability” language to “cross-cohort evaluation” to match the actual estimand.

Location in revised materials: Clean manuscript p. 1, lines 1-14; submission package filenames.

Journal requirement 2

The Data Availability Statement should provide the repository name and DOI/accession number or direct link for each dataset.

Response: The Data and Code Availability section now names the NCBI Gene Expression Omnibus and lists every accession used in the reported analyses: GSE25055, GSE25065, GSE32646, GSE163882, GSE194040, GSE319641, and GSE176078, with GSE20194 identified as an excluded overlapping accession. S2 additionally names the decisive public processed matrix files and platform annotation files.

Location in revised materials: Data and Code Availability, pp. 11-12, lines 246-252; S2 INPUT_MANIFEST.tsv and PREPROCESSING_PROTOCOL.md.

Journal requirement 3

Please upload new copies of Figures 1 to 5 because the detail is not clear.

Response: All five main figures were regenerated from source tables. The journal-supplied files are 600-dpi RGB TIFFs without alpha channels, use LZW compression, remain within the 7.5-inch maximum width and 10-MB file-size limit, and use figure text at 8 pt or larger. Figure 1 was simplified to separate the primary cohort path from the GSE20194 overlap audit; Figures 2-5 were similarly regenerated with larger labels and the revised evidence hierarchy.

Location in revised materials: Fig 1 caption, p. 12, lines 261-267; Fig 2 caption, p. 13, lines 283-287; Fig 3 caption, p. 15, lines 320-324; Fig 4 caption, p. 16, lines 339-341; Fig 5 caption, p. 16, lines 356-362; package Figures/Fig1.tif through Fig5.tif; figure source tables are included in S2 File under results/figure_source/.

Journal requirement 4

Evaluate any reviewer-recommended citations for relevance; citation is not mandatory unless directed by the editor.

Response: No reviewer required citation of a specific paper. In response to Reviewer 2's request for a clearer biological rationale, we added one directly relevant TNBC study showing adaptive mitochondrial oxidative-phosphorylation/fusion changes in chemotherapy-surviving TNBC models, while explicitly avoiding a causal claim for the selected chaperone genes. Other references remain focused on the clinical endpoint, public cohorts, MitoCarta pathway definition, reporting standards, meta-analytic methods, tissue-composition methods, NeoTRIP, and single-cell source data.

Location in revised materials: Biological rationale, p. 3, lines 53-59; reference 4, p. 20.

Additional Editor requirement: point-by-point response

Provide a detailed point-by-point response addressing every comment and specify exact page and line numbers.

Response: This document responds to each numbered and unnumbered substantive comment below. Page/line references correspond to the clean revised manuscript with continuous line numbering.

Location in revised materials: clean revised manuscript with final continuous line numbering; supporting files as cited in this response.

Additional Editor requirement: clean and marked-up files

Upload both a clean revised manuscript and a marked-up copy highlighting all modifications.

Response: We provide a clean revised manuscript and a marked-up Word comparison generated from the original submitted manuscript against the final clean revision. The marked file shows the original submitted text as deletions and the revised text as insertions; accepting all revisions yields the final clean manuscript, and rejecting all revisions restores the original submitted substantive manuscript.

Location in revised materials: clean revised manuscript with final continuous line numbering; supporting files as cited in this response.

Additional Editor requirement: data and code integrity

Ensure underlying data, cohort definitions, and scaling code are clearly documented and accessible.

Response: S2 provides patient-level derived data, named public processed inputs, preprocessing documentation, and executable workflows for the core cohort models, RankScore, source-level LOCO analysis, patient-level I-SPY2 and reduced-input single-cell analyses, together with machine-readable NeoTRIP outputs and historical NeoTRIP generator code that requires the original project inputs.

Location in revised materials: Data and Code Availability, pp. 11-12, lines 246-252; S2 README.md, INPUT_MANIFEST.tsv, PREPROCESSING_PROTOCOL.md, ANALYSIS_CHANGELOG.md, ANALYTICAL_HISTORY.md, code/, results/, and the contextual-analysis documentation described in the S2 README.

Reviewer #1

Comment 1

In the abstract, the authors need to mention the improvement of the proposed model as compared to the existing models.

Response: The study evaluates a fixed candidate score rather than developing a new predictive model. The Abstract now states the fixed-score estimand, the four-cohort and three-source results, the fixed RankScore sensitivity, and the study-source precision directly. Model-improvement metrics would address a different model-development question.

Location in revised materials: Abstract, p. 2, lines 15-39.

Comment 2

At the introduction, the key contributions of the proposed model should be concise and clearly provided.

Response: We revised the Introduction to state the knowledge gap and contribution concisely: public accession labels do not guarantee participant independence, cohort-specific normalization creates reference dependence, and the paper evaluates one fixed mitochondrial score as a test case rather than proposing a new model.

Location in revised materials: Introduction, p. 3, lines 40-64.

Comment 3

What are the limitations of the proposed study?

Response: The Discussion now consolidates the principal limitations: only three principal study sources, heterogeneity among public studies, retrospective design, one small cohort, proxy tissue-composition measures, reference dependence, no linked NeoTRIP clinical outcomes in the available data, and only eight informative single-cell pairs.

Location in revised materials: Discussion, pp. 17-19, lines 368-425.

Comment 4

The authors need to compare the proposed model with the existing state-of-the-art models.

Response: A state-of-the-art prediction benchmark was not added because the manuscript evaluates a fixed candidate score rather than developing a new prediction model. The claim remains fixed-score association and cross-source reproducibility, distinct from deployable prediction.

Location in revised materials: Conclusions, p. 19, lines 426-432.

Comment 5

What are the future directions of the proposed study?

Response: We added a focused future-validation paragraph. Stronger validation requires genuinely independent study populations, a fixed single-sample scoring implementation or external reference distribution, prospectively specified preprocessing/analysis rules, more independent study sources, and prespecified treatment-interaction analysis for treatment-selection claims.

Location in revised materials: Future-validation statement, p. 18, lines 411-414.

Comment 6

The authors need to revise the figures in 300 dpi for clear visualization.

Response: All main figures were regenerated and are supplied as 600-dpi, RGB, LZW-compressed TIFFs meeting the journal size and width limits. Labels and panel structure were simplified, and all explicit figure text is 8 pt or larger.

Location in revised materials: Fig 1 caption, p. 12, lines 261-267; Fig 2 caption, p. 13, lines 283-287; Fig 3 caption, p. 15, lines 320-324; Fig 4 caption, p. 16, lines 339-341; Fig 5 caption, p. 16, lines 356-362.

Reviewer #2

Comment 1

GSE25055 and GSE25065 originated from the same parent study. Please justify treating them as separate cohorts in the primary meta-analysis and explain the effect on independence.

Response: We retained the submitted four-accession analysis as primary to avoid changing the primary estimand after viewing results, but now make the dependence explicit. GSE25055 and GSE25065 retain separate accession-specific score references/intercepts; a main-text sensitivity combines them into one Hatzis/MD Anderson source effect using separate accession intercepts and one shared score slope, then pools that effect with GSE32646 and GSE163882. The three-source result is reported alongside, but is not called co-primary.

Location in revised materials: Study design/estimand, p. 4, lines 73-79; source-structure meta-analysis Methods, p. 8, lines 165-169; Results, p. 13, lines 278-292; Discussion, p. 18, lines 399-404; S1 Table S4.

Comment 2

The score was calculated using within-cohort standardization. Please explain why this method was selected and discuss its limitations.

Response: As noted above, the submitted reference-scope wording was corrected to match the executed analysis: target genes were standardized across the full processed matrix for each accession, followed by composite re-standardization in the analysis cohort. We added the mathematical definition and a single-sample RankScore sensitivity to assess dependence on cross-patient standardization.

Location in revised materials: Score construction, pp. 6-7, lines 121-150; RankScore Methods, pp. 8-9, lines 173-185; RankScore Results, p. 14, lines 296-302; Discussion, p. 17, lines 381-388; S2 PREPROCESSING_PROTOCOL.md, ANALYSIS_CHANGELOG.md, and code/02_rebuild_fixed_score_from_public_processed.py.

Comment 3

The score correlated strongly with proliferation, while adjusted analyses were significant although the raw score was not. Please explain and provide correlations with the other adjustment variables.

Response: The Results report the primary estimate first and distinguish direct adjustment, same-cohort residualization, and LOCO residualization as different estimands. Score–proliferation correlations are reported for every primary cohort, immune/stromal relationships are summarized across cohorts, and the full cohort-specific correlation matrix is shown in S1 Fig S3 with machine-readable tables in S2. Held-out LOCO residual correlations with proliferation, immune, and stromal variables quantify transport of the nuisance relationships.

Location in revised materials: Results, pp. 14-15, lines 303-328; Discussion, p. 18, lines 389-398; S1 Figs S3-S4 and S2 correlation tables.

Comment 4

The LOCO residualization procedure is difficult to follow. Please provide a clearer step-by-step explanation, preferably with a schematic figure or workflow.

Response: We rewrote LOCO as a stepwise training/held-out procedure: estimate nuisance slopes in the other cohorts with training-cohort intercepts, compute the training residual SD, apply the training-derived nuisance relation to the held-out cohort, express the held-out residual in training-SD units, fit a residual-only held-out pCR model, and pool fold effects. We also report held-out scale transfer and residual–background correlations in S1 Fig S4. The main Figure 1 shows the analysis hierarchy so LOCO is visually separated from the primary and direct adjusted models.

Location in revised materials: LOCO Methods, pp. 9-10, lines 195-208; Results, p. 15, lines 313-328; Fig 3 caption, p. 15, lines 320-324; S1 Fig S4.

Comment 5

The authors set negative ComBat-adjusted values to zero before log transformation. Please provide a stronger justification for this approach.

Response: As noted above, the submitted NeoTRIP description was corrected to match the executed raw-score analysis, which used the provider-supplied linear-scale matrix and all 401 profiles as the standardization reference. All 15 target-gene values were nonnegative in this matrix, so clipping is not relevant to the raw-score calculation. S2 documents the workflow and reported outputs.

Location in revised materials: NeoTRIP Methods, p. 10, lines 209-216; Results, p. 16, lines 335-345; Fig 4 caption, p. 16, lines 339-341; Discussion, p. 18, lines 393-398; S1 Table S9; S2 PREPROCESSING_PROTOCOL.md, ANALYSIS_CHANGELOG.md, and recovered_workflows/neotrip/.

Comment 6

Please provide a clear table comparing patient characteristics, treatment regimens, sample processing, and pCR definitions across the primary cohorts.

Response: We added a main-text cohort table reporting parent study/source, platform and pretreatment specimen, neoadjuvant treatment, retained pCR definition, and n/pCR. Unreported pathology details were left unspecified. Age, stage, and related patient-level variables were not consistently available in harmonizable form across all four public cohort inputs and were not imputed for cross-cohort comparison.

Location in revised materials: Cohort eligibility/characteristics Methods, pp. 4-5, lines 80-100; Table 1 and note, p. 5, lines 89-94; S1 Table S1; S2 INPUT_MANIFEST.tsv and PREPROCESSING_PROTOCOL.md.

Comment 7

Please clearly report the definition of pCR used in each dataset and discuss differences.

Response: The Methods and Table 1 now retain source-study definitions rather than retrospectively harmonizing them. GSE32646 required no residual invasive cancer in breast and axilla; GSE163882 used the pathology-report definition from the contributing institution; for GSE25055/GSE25065, the accessible source report did not support inferring a uniform nodal/DCIS rule. The limitation of non-uniform definitions is stated explicitly.

Location in revised materials: Cohort eligibility and source-defined pCR Methods, pp. 4-5, lines 80-94; Discussion limitations, p. 19, lines 415-425.

Comment 8

Please provide more detail about how the final 15 genes were selected from the mitochondrial proteostasis/chaperone pathway and why other genes were excluded.

Response: We now identify the parent 16-gene MitoCarta3.0 Chaperones pathway, list all 15 retained genes, specify the exact minimum 80% coverage scope across GPL96, GPL570, and the I-SPY2 expression platform, specify alias handling, and state that DNLZ was excluded because GPL96 lacked a reliable probe. GSE163882 was incorporated after confirming measurability of the 15 target genes. Equal positive weights and the no-cutpoint rule are also explicit.

Location in revised materials: Pathway definition and score construction, pp. 6-7, lines 121-150; S2 PREPROCESSING_PROTOCOL.md and INPUT_MANIFEST.tsv.

Comment 9

The single-cell analysis includes few tumors. Please provide more details on malignant/non-malignant identification and pseudobulk analysis and emphasize the small sample size.

Response: The analysis uses the processed single-cell inputs with source-provided patient field `orig.ident` and cell-type annotations; `Cancer Epithelial` is the malignant-epithelial label. Fig 5a retains patient×major-cell-type groups with at least 20 cells for display. Fig 5b eligibility requires one `Cancer Epithelial` row plus at least one non-cancer row with positive pooled counts. Eight of the 10 TNBC patients met that rule; CID3946 and CID44041 did not. Non-cancer counts are pooled within patient before CP10K, log1p, and 15-gene averaging. Patients—not cells—are the inferential units.

Location in revised materials: Single-cell Methods, pp. 10-11, lines 217-232; Results, p. 16, lines 346-362; Discussion, p. 18, lines 393-398 and pp. 19, lines 415-425; S1 Table S10.

Comment 10

Please provide a more complete workflow from the original public datasets to the final results.

Response: S2 now contains a public-input manifest, preprocessing protocol, patient-level derived analysis tables, platform-specific primary-score reconstruction and core-model workflows, source-structure meta-analysis, RankScore, single-cell analysis, figure regeneration, environment files, and hashes. The named public processed matrices and platform-annotation resources are listed in INPUT_MANIFEST.tsv.

Location in revised materials: Statistical software/reproducibility and Data and Code Availability, pp. 11-12, lines 233-252; S2 README.md, INPUT_MANIFEST.tsv, PREPROCESSING_PROTOCOL.md, code/, results/, SHA256SUMS.txt, and recovered_workflows/.

Comment 11

Please state the specific knowledge gap more clearly in the Introduction.

Response: The revised Introduction now frames two linked gaps: accession labels do not guarantee participant independence, and cohort-specific normalization can yield a reproducibly calculated but reference-dependent score. The study then evaluates a fixed score as a test case for limited cross-cohort association/reproducibility.

Location in revised materials: Introduction, p. 3, lines 47-64.

Comment 12

The biological rationale for selecting a mitochondrial proteostasis-associated score in relation to TNBC response/pCR requires further explanation.

Response: We expanded the biological bridge. The Introduction notes that chemotherapy-surviving TNBC models can show adaptive increases in mitochondrial oxidative phosphorylation and fusion, while MitoCarta defines mitochondrial chaperone/protein-quality-control functions. Mitochondrial proteostasis therefore provides a biologically plausible stress-response context for testing the fixed score.

Location in revised materials: Introduction, p. 3, lines 53-59; reference 4, p. 20.

Comment 13

Please clarify whether the primary objective is to evaluate a specific mitochondrial gene score or demonstrate methodological challenges in public transcriptomic validation.

Response: The primary objective is now stated explicitly as estimating the association between the fixed cohort-relative 15-gene score and source-study-defined pCR, with consistency assessed across study sources. Methodological issues such as overlap, reference dependence, and specimen context are interpreted as factors affecting that score evaluation rather than as a separate model-development objective.

Location in revised materials: Abstract, p. 2, lines 15-39; Introduction, p. 3, lines 60-64; primary estimand, p. 4, lines 73-79.

Comment 14

Please provide more detail about how duplicate patients were identified and verified.

Response: The matching process is now explicit. Matching was deterministic on source patient identifiers; receptor annotation, pCR concordance, and expression similarity were secondary verification checks and were not used to select matches. Three R1 repeats were collapsed, 58 IDs were present in GSE25055, 57 were strict TNBC in both accessions, M180 was receptor-discordant, and 10 had no matching identifier. The unmatched residual subset was not analyzed as a separate cohort.

Location in revised materials: Patient-overlap Methods, p. 6, lines 101-120; Results, p. 12, lines 254-277; Fig 1 caption, p. 12, lines 261-267; S1 Table S3; S2 data/overlap/ and protocol/01_Overlap_Audit_Prespecified.md.

Comment 15

Please justify random-effects meta-analysis with few cohorts and provide sensitivity using alternative approaches/estimators.

Response: We retained REML with modified Hartung-Knapp inference as the submitted primary procedure but now explicitly handle the tau²=0 boundary, report tau² and I² descriptively, and state that I²=0% is not proof of homogeneity. We added a fixed Paule–Mandel tau² sensitivity and the three-source sensitivity. The three-source interval is substantially wider and is reported alongside the primary result.

Location in revised materials: Meta-analysis/source-sensitivity Methods, p. 8, lines 160-169; Results, p. 13, lines 278-292; Discussion, p. 18, lines 399-404; S1 Table S4.

Comment 16

Please strengthen the justification/limitations of within-cohort z-score normalization and, if possible, provide an alternative scoring sensitivity.

Response: We now state that the cohort-relative score depends on accession-level and cohort-level reference distributions and therefore is not directly deployable for a new individual without a reference. A single fixed within-sample RankScore sensitivity was implemented using the same 15 genes and a fixed 12,151-gene background universe. The algorithm, universe, gene directions, cutoff rule, and cohort set were specified before evaluating the association.

Location in revised materials: Score construction, p. 7, lines 136-150; RankScore Methods, pp. 8-9, lines 173-185; Results, p. 14, lines 296-302; Discussion, p. 17, lines 381-388; S1 Table S5 and Fig S2; S2 code/04_rebuild_rank_score_from_public_processed.py.

Comment 17

Please describe preprocessing by platform, including normalization, probe-to-gene mapping, multiple probes, and missing genes; a summary table would improve reproducibility.

Response: We expanded both the manuscript and S2. Table 1 reports platform/specimen context. The score method specifies unambiguous symbol mapping, fixed aliases, within-sample median aggregation for multiple retained microarray probes and, for GSE163882, log2(TPM + 1) transformation followed by within-sample maximum aggregation of duplicate symbols, the platform-coverage rule, and the reference distributions. S2 INPUT_MANIFEST.tsv identifies the public processed expression matrices and platform-annotation resources by dataset and the packaged derived mapping tables. PREPROCESSING_PROTOCOL.md summarizes the score reference scopes and preprocessing provenance, while code/02_rebuild_fixed_score_from_public_processed.py implements the array probe-to-gene mapping, RNA-seq gene-level processing, alias handling, and score-reconstruction steps from the named public processed inputs.

Location in revised materials: Table 1 and note, p. 5, lines 89-94; pathway/score construction, pp. 6-7, lines 121-150; Data and Code Availability, pp. 11-12, lines 246-252; S2 INPUT_MANIFEST.tsv, PREPROCESSING_PROTOCOL.md, and code/02_rebuild_fixed_score_from_public_processed.py.

Comment 18

The final paragraph of the Introduction contains substantial methodological detail. Please simplify it and focus on the research question and objectives.

Response: We shortened the Introduction and moved technical implementation to the Methods. The final paragraph now states the fixed-score pCR question and the broad analysis components without enumerating model details.

Location in revised materials: Final Introduction paragraph, p. 3, lines 60-64.

Reviewer #3

Major comment 1

Clarify the precise estimand and intended claim; distinguish fixed-score association/cross-cohort reproducibility from clinical transportability or a deployable assay.

Response: We agree and revised the framing throughout. The Methods define the primary estimand as the association between the fixed cohort-relative score and source-study-defined pCR, summarized across four accession-defined cohorts. Clinical deployment and treatment-effect prediction are outside that estimand. “Transportability” language implying clinical deployability was removed from the title and conclusions.

Location in revised materials: Abstract, p. 2, lines 15-39; Study design/estimand, p. 4, lines 66-79; Conclusions, p. 19, lines 426-432.

Major comment 2

The effective number of independent study sources is very small; distinguish patients, accessions/cohorts, and study sources and avoid broad external-validation language.

Response: We now report the hierarchy explicitly: 285 unique patients, four accession-defined cohorts, three principal study sources. GSE25055 and GSE25065 are treated as separate accessions in the primary analysis but one source in the source-structure sensitivity. The small number of sources is emphasized as a central limitation of precision and generalizability.

Location in revised materials: Abstract, p. 2, lines 20-37; Study design/estimand, p. 4, lines 73-79; Results, pp. 12-13, lines 268-292; Discussion, p. 18, lines 399-404; S1 Tables S1/S2/S4.

Major comment 3

Provide stronger justification for random-effects meta-analysis with four cohorts, report tau², and do not interpret I²=0% as absence of heterogeneity.

Response: The revised Methods compare the REML tau²=0 boundary with the numerical optimum, use mKH inference, report I² descriptively, and add Paule-Mandel tau² sensitivity. The primary four-cohort tau² is 0 after boundary comparison; the three-source REML tau² is 0.0201 and PM tau² is 0.00717. With only three to four analysis units, the manuscript reports effect estimates and confidence intervals without treating I²=0% as evidence of homogeneity.

Location in revised materials: Meta-analysis/source-sensitivity Methods, p. 8, lines 160-169; Results, p. 13, lines 278-292; Discussion, p. 18, lines 399-404; S1 Table S4.

Major comment 4

The exploratory adjusted associations require especially cautious interpretation; make them subordinate to the primary null result and clarify LOCO.

Response: We reorganized Results so the primary and source-level findings appear first. The adjusted/residual section labels the nominal findings as exploratory decomposition. LOCO is defined as a different estimand that tests whether nuisance relationships learned in other cohorts transport to the held-out cohort.

Location in revised materials: Primary/direct-adjustment Methods, p. 8, lines 151-172; LOCO Methods, pp. 9-10, lines 195-208; Table 2, p. 14, line 293; adjusted/LOCO Results, pp. 14-15, lines 303-328; Discussion, p. 18, lines 389-392.

Major comment 5

The prior use of the same cohorts and outcomes introduces analytical history. Clarify when the 15-gene score was specified, prior related pCR analyses, changes after viewing pCR, and whether the internal record can be public.

Response: The Methods now describe the 15-gene specification as fixed before the present score-specific pCR analysis, following related pCR analyses with a different mitochondrial gene set in overlapping cohorts. The Discussion explicitly acknowledges the resulting potential analytical flexibility; this retrospective specification was not a public preregistration or fully outcome-blinded development. The RankScore sensitivity was added during revision, with its implementation defined before evaluating its association with pCR. S2 includes the analytical-history record and revision protocol.

Location in revised materials: Pathway definition and score construction, pp. 6-7, lines 121-150; Discussion limitations, p. 19, lines 415-425; S2 ANALYTICAL_HISTORY.md, ANALYSIS_CHANGELOG.md, and protocol/.

Major comment 6

Describe the patient-overlap matching procedure in greater detail, including identifiers, formatting, secondary confirmation, and uncertain matches.

Response: We corrected and expanded the overlap audit. Matching is based on deterministic source patient identifiers; receptor annotations, pCR concordance, and expression similarity are secondary verification checks. Three repeat records were collapsed, 58 IDs were present in GSE25055, 57 were strict TNBC in both, M180 was receptor-discordant, and 10 were unmatched. The unmatched residual subset was not analyzed as a separate cohort. The full mapping/counterfactual tables are in S2/S1.

Location in revised materials: Patient-overlap Methods, p. 6, lines 101-120; Results, p. 12, lines 254-277; Fig 1 caption, p. 12, lines 261-267; S1 Table S3; S2 data/overlap/ and protocol/01_Overlap_Audit_Prespecified.md.

Major comment 7

The cohort-relative standardization affects interpretation. Add a mathematical definition and, if possible, a cohort-independent/rank sensitivity.

Response: We added the requested mathematical definition and corrected the submitted reference-scope wording as described above. The equation makes the accession-level and analysis-cohort reference distributions explicit. We also added one fixed single-sample RankScore sensitivity using the same 15 genes and a fixed 12,151-gene background universe.

Location in revised materials: Primary score construction, p. 7, lines 136-150; RankScore Methods, pp. 8-9, lines 173-185; Results, p. 14, lines 296-302; Discussion, p. 17, lines 381-388; S2 PREPROCESSING_PROTOCOL.md, ANALYSIS_CHANGELOG.md, code/02_rebuild_fixed_score_from_public_processed.py, and code/04_rebuild_rank_score_from_public_processed.py.

Major comment 8

NeoTRIP preprocessing requires a non-clipping sensitivity or stronger justification.

Response: The submitted NeoTRIP description was corrected as described above. The executed raw-score analysis used the provider-supplied linear-scale matrix and all 401 profiles as the standardization reference; all 15 target-gene values were nonnegative. The background-feature decompositions remain contextual rather than a primary mechanistic test.

Location in revised materials: NeoTRIP Methods, p. 10, lines 209-216; Results, p. 16, lines 335-345; Fig 4 caption, p. 16, lines 339-341; Discussion, p. 18, lines 393-398; S1 Table S9; S2 PREPROCESSING_PROTOCOL.md, ANALYSIS_CHANGELOG.md, and recovered_workflows/neotrip/.

Major comment 9

The single-cell analysis is informative but underpowered; eight paired patients should be treated as hypothesis-generating.

Response: We treat the single-cell analysis as contextual and patient-level. The analysis uses the 10-TNBC subset of GSE176078 and source-provided `orig.ident` and cell-type annotations; `Cancer Epithelial` is the malignant epithelial label used here. Fig 5b eligibility requires one `Cancer Epithelial` row plus non-cancer rows with positive pooled counts. Eight patients met that rule; CID3946 and CID44041 did not. S2 includes the reduced cell-level inputs and executable workflow, including the cycling-state sensitivity.

Location in revised materials: Single-cell Methods, pp. 10-11, lines 217-232; Results, p. 16, lines 346-362; Discussion, p. 18, lines 393-398 and pp. 19, lines 415-425; S1 Table S10; S2 recovered_workflows/single_cell/.

Major comment 10

Distinguish absence of evidence from evidence of absence.

Response: The Abstract, Discussion, and Conclusion now state the result directly: neither fixed implementation showed a reproducible cross-cohort association, and the study-source estimates remained imprecise. The wide intervals indicate limited precision around the effect estimate.

Location in revised materials: Abstract, p. 2, lines 27-37; Results, pp. 13-14, lines 278-302; Discussion, pp. 17-18, lines 368-404; Conclusions, p. 19, lines 426-432.

Major comment 11

Keep I-SPY2 clearly separate from the primary TNBC analysis and avoid implying that it validates the score.

Response: I-SPY2 remains contextual evidence, not a strict-TNBC primary cohort. The patient-level I-SPY2 analysis includes treatment-arm-adjusted, arm+MammaPrint, and score-by-arm interaction models. Because I-SPY2 uses HR-negative/HER2-negative eligibility and a multi-arm treatment structure, those analyses remain outside the primary four-cohort meta-analysis.

Location in revised materials: I-SPY2 source boundary, p. 5, lines 95-100; Results, p. 17, lines 363-367; Discussion, p. 18, lines 405-410; S1 Table S11.

Major comment 12

Retain the distinction between response association and treatment prediction.

Response: The manuscript distinguishes response association from treatment-effect prediction. Treatment-selection claims require prespecified, reproducible score-by-treatment interaction evidence.

Location in revised materials: I-SPY2 Discussion, p. 18, lines 405-410; future-validation statement, p. 18, lines 411-414.

Data availability and reproducibility

Provide complete preprocessing scripts or a sufficiently detailed step-by-step preprocessing protocol, and ensure S2 contains all variables needed to reproduce every primary estimate.

Response: S2 now addresses this requirement. Core estimates can be re-fitted from the 285-patient derived table; score construction is traceable to explicitly named public processed matrices and platform annotations; the fixed RankScore workflow enforces the 12,151-gene universe by gene identity; and the source-level LOCO diagnostic is executable from the 285-patient table. S2 also supplies executable refits for patient-level I-SPY2 models and a reduced-input single-cell workflow. For NeoTRIP, S2 provides derived tables, outputs, historical generator code, and archived checks; the generator requires the original project inputs and path configuration.

Location in revised materials: Statistical software/reproducibility and Data and Code Availability, pp. 11-12, lines 233-252; S2 README.md, INPUT_MANIFEST.tsv, PREPROCESSING_PROTOCOL.md, code/, results/, SHA256SUMS.txt, and recovered_workflows/.

Statistical reporting

Report cohort events/non-events and model n; tau²; correlation confidence-interval methods; small-sample corrections; the complete exploratory analysis set/multiplicity; and acknowledge limited power.

Response: These items are documented in the revision. Cohort/model n and pCR events are reported in the main Methods and S1 Table S2; tau² is reported for primary and sensitivity meta-analyses; S1 Supplementary Methods specifies Fisher-z correlation intervals and Fisher-z random-effects pooling; it also states that ordinary binomial GLM/Wald intervals were used, no Firth penalty was applied, and all retained core fits converged without complete separation. S1/S2 inventory the exploratory families and FDR procedures. The Discussion emphasizes the small number of independent study sources and the resulting imprecision.

Location in revised materials: Results, pp. 13-16, lines 278-362; Discussion, pp. 18-19, lines 399-425; S1 Supplementary Methods and Tables S2, S4, S12; S2 results/.

Presentation and language

Shorten complex methodological sentences and ensure consistent terminology for cohort, accession, study source, patient, record, and sample.

Response: The Methods were reorganized into topic-specific subsections, and terminology was standardized. “Patient” refers to the biological participant, “record/aliquot” to observations that may duplicate a patient, “accession-defined cohort” to the analytic dataset, and “study source” to the parent population/source. These terms are used consistently throughout the revised manuscript.

Location in revised materials: Reorganized Methods, pp. 4-12, lines 65-252; patient/accession/source terminology in overlap and source-structure sections, pp. 6-8, lines 101-168 and pp. 12-13, lines 254-292.

Minor comment 1

Define LOCO and computational abbreviations at first use.

Response: LOCO is now introduced as leave-one-cohort-out before abbreviation, and key computational terms are expanded or described in context.

Location in revised materials: LOCO Methods, pp. 9-10, lines 195-208; Results, p. 15, lines 313-328; S2 code/08_rebuild_leave_one_source_out.py.

Minor comment 2

Distinguish patients, samples, aliquots, and records.

Response: The overlap/aliquot sections now use these terms explicitly and separately; all clinical analyses use one row per patient after the GSE163882 aliquot collapse.

Location in revised materials: overlap Methods, p. 6, lines 101-120; overlap Results, p. 12, lines 254-277.

Minor comment 3

Provide a transparent flow diagram showing records, overlap, aliquot handling, eligibility, and final analysis numbers.

Response: Figure 1 was redesigned as a two-path flow diagram separating the primary candidate/deduplication path from the GSE20194 overlap audit and descriptive counterfactual.

Location in revised materials: Fig 1 caption, p. 12, lines 261-267; S1 Table S3.

Minor comment 4

Explain the treatment of unmatched GSE20194 records and why the entire accession was excluded.

Response: The revised audit identifies 10 GSE20194 patients with no matching GSE25055 identifier. Because participant reuse was extensive, GSE20194 was excluded as an independent primary cohort; the unmatched residual subset was not analyzed as a separate cohort.

Location in revised materials: overlap Methods, p. 6, lines 101-120; overlap Results, p. 12, lines 254-277.

Minor comment 5

Clarify that this is a computational secondary analysis and not wet-laboratory experimental validation.

Response: The Study Design section identifies the work as a retrospective secondary analysis of publicly accessible, de-identified transcriptomic and clinical data. The manuscript does not present the study as a new experimental or wet-laboratory validation.

Location in revised materials: Study design and reporting, p. 4, lines 66-72; Statistical software/reproducibility, p. 11, lines 233-242.

Minor comment 6

Ensure all substantive methodological/statistical decisions remain attributable to the investigators rather than AI-assisted suggestions.

Response: All substantive methodological and statistical decisions were made by the investigators. OpenAI ChatGPT was used for language editing, code review, and consistency checking. The authors are responsible for all data, analyses, results, and manuscript content.

Location in revised materials: Use of generative artificial intelligence, p. 11, lines 243-245.

Reviewer #4

Overall recommendation

Reviewer #4 recommended acceptance and provided no additional requested changes.

Response: We thank Reviewer 4 for the free-text recommendation to accept the manuscript and note that no specific revision requests were provided. We addressed the Editor and other reviewers in detail.

Location in revised materials: clean revised manuscript with final continuous line numbering; supporting files as cited in this response.

Attachment Submitted filename: Response_to_Reviewers.docx

# Peer review: Decision Letter 1

**DOI:** 10.1371/journal.pone.0355652.r003

**Authors:** UDAYAN BHATTACHARYA

27 Sep 2026

Patient overlap, cohort-relative scaling, and biological context in cross-cohort evaluation of a mitochondrial proteostasis-associated score in triple-negative breast cancer

PONE-D-26-36973R1

Dear Dr. Weng,

We’re pleased to inform you that your manuscript has been judged scientifically suitable for publication and will be formally accepted for publication once it meets all outstanding technical requirements.

Within one week, you’ll receive an e-mail detailing the required amendments. When these have been addressed, you’ll receive a formal acceptance letter and your manuscript will be scheduled for publication.

An invoice will be generated when your article is formally accepted. Please note, if your institution has a publishing partnership with PLOS and your article meets the relevant criteria, all or part of your publication costs will be covered. Please make sure your user information is up-to-date by logging into Editorial Manager at Editorial Manager® and clicking the ‘Update My Information' link at the top of the page. For questions related to billing, please contact billing support.

If your institution or institutions have a press office, please notify them about your upcoming paper to help maximize its impact. If they’ll be preparing press materials, please inform our press team as soon as possible -- no later than 48 hours after receiving the formal acceptance. Your manuscript will remain under strict press embargo until 2 pm Eastern Time on the date of publication. For more information, please contact onepress@plos.org.

Kind regards,

UDAYAN BHATTACHARYA

Academic Editor

PLOS One

Additional Editor Comments (optional):

Dear Dr. Weng,

Thank you for re-submitting your manuscript, "Patient overlap, cohort-relative scaling, and biological context in cross-cohort evaluation of a mitochondrial proteostasis-associated score in triple-negative breast cancer" (PONE-D-26-36973R1), to PLOS ONE.

We are pleased to inform you that your manuscript has now been accepted for publication in PLOS ONE.

The revisions made in response to the reviewers' comments have adequately addressed the concerns raised during the initial review process. Thank you for your thorough point-by-point response and for revising the manuscript to meet PLOS ONE’s publication standards.

Our publishing team will be in touch with you shortly regarding the final production process, page proofs, and publication details.

Congratulations on the publication of your work.

Sincerely,

Dr. Udayan Bhattacharya

Academic Editor, PLOS ONE

Reviewers' comments:

Reviewer's Responses to Questions

**Comments to the Author**

Reviewer #1: All comments have been addressed

**********

2. Is the manuscript technically sound, and do the data support the conclusions?

Reviewer #1: Yes

**********

3. Has the statistical analysis been performed appropriately and rigorously?

Reviewer #1: Yes

**********

4. Have the authors made all data underlying the findings in their manuscript fully available?

The PLOS Data policy

Reviewer #1: Yes

**********

5. Is the manuscript presented in an intelligible fashion and written in standard English?

Reviewer #1: Yes

**********

Reviewer #1: The required comments are successfully addressed . Therefore, the paper has significantly improved. From my side the paper can be accepted

**********

what does this mean?). If published, this will include your full peer review and any attached files.

If you choose “no”, your identity will remain anonymous but your review may still be made public.

**Do you want your identity to be public for this peer review?** For information about this choice, including consent withdrawal, please see our Privacy Policy

Reviewer #1: No

**********

# Editor report: Acceptance letter

**DOI:** 10.1371/journal.pone.0355652.r004

**Authors:** UDAYAN BHATTACHARYA

PONE-D-26-36973R1

PLOS One

Dear Dr. Weng,

I'm pleased to inform you that your manuscript has been deemed suitable for publication in PLOS One. Congratulations! Your manuscript is now being handed over to our production team.

At this stage, our production department will prepare your paper for publication. This includes ensuring the following:

* All references, tables, and figures are properly cited

* All relevant supporting information is included in the manuscript submission,

* There are no issues that prevent the paper from being properly typeset

You will receive further instructions from the production team, including instructions on how to review your proof when it is ready. Please keep in mind that we are working through a large volume of accepted articles, so please give us a few days to review your paper and let you know the next and final steps.

Lastly, if your institution or institutions have a press office, please let them know about your upcoming paper now to help maximize its impact. If they'll be preparing press materials, please inform our press team within the next 48 hours. Your manuscript will remain under strict press embargo until 2 pm Eastern Time on the date of publication. For more information, please contact onepress@plos.org.

You will receive an invoice from PLOS for your publication fee after your manuscript has reached the completed accept phase. If you receive an email requesting payment before acceptance or for any other service, this may be a phishing scheme. Learn how to identify phishing emails and protect your accounts at https://explore.plos.org/phishing.

If we can help with anything else, please email us at customercare@plos.org.

Thank you for submitting your work to PLOS One and supporting open access.

Kind regards,

PLOS One Editorial Office Staff

on behalf of

Dr. UDAYAN BHATTACHARYA

Academic Editor

PLOS One
